# BDH1‐mediated LRRC31 regulation dependent on histone lysine β‐hydroxybutyrylation to promote lung adenocarcinoma progression

**DOI:** 10.1002/mco2.449

**Published:** 2023-12-13

**Authors:** Jingjing Huang, Lu Liang, Shiyao Jiang, Yueying Liu, Hua He, Xiaoyan Sun, Yi Li, Li Xie, Yongguang Tao, Li Cong, Yiqun Jiang

**Affiliations:** ^1^ The Key Laboratory of Model Animal and Stem Cell Biology in Hunan Province, Hunan Normal University Changsha Hunan China; ^2^ Department of Basic Medicine, School of Medicine, Hunan Normal University Changsha Hunan China; ^3^ Department of Head and Neck Surgery Hunan Cancer Hospital, Xiangya School of Medicine, Central South University Changsha Hunan China; ^4^ Department of Pathology Key Laboratory of Carcinogenesis and Cancer Invasion, Ministry of Education, Xiangya Hospital, School of Basic Medicine, Central South University Changsha Hunan China

**Keywords:** BDH1, H3K9bhb, LRRC31, lung adenocarcinoma, β‐hydroxybutyrate

## Abstract

Lung adenocarcinoma (LUAD) is the most common form of lung cancer, with a consistently low 5‐year survival rate. Therefore, we aim to identify key genes involved in LUAD progression to pave the way for targeted therapies in the future. BDH1 plays a critical role in the conversion between acetoacetate and β‐hydroxybutyrate. The presence of β‐hydroxybutyrate is essential for initiating lysine β‐hydroxybutyrylation (Kbhb) modifications. Histone Kbhb at the H3K9 site is attributed to transcriptional activation. We unveiled that β‐hydroxybutyrate dehydrogenase 1 (BDH1) is not only conspicuously overexpressed in LUAD, but it also modulates the overall intracellular Kbhb modification levels. The RNA sequencing analysis revealed leucine‐rich repeat‐containing protein 31 (LRRC31) as a downstream target gene regulated by BDH1. Ecologically expressed BDH1 hinders the accumulation of H3K9bhb in the transcription start site of LRRC31, consequently repressing the transcriptional expression of LRRC31. Furthermore, we identified potential BDH1 inhibitors, namely pimozide and crizotinib, which exhibit a synergistic inhibitory effect on the proliferation of LUAD cells exhibiting high expression of BDH1. In summary, this study elucidates the molecular mechanism by which BDH1 mediates LUAD progression through the H3K9bhb/LRRC31 axis and proposes a therapeutic strategy targeting BDH1‐high‐expressing LUAD, providing a fresh perspective for LUAD treatment.

## Graphical abstract

BDH1 promotes LUAD development by regulating LRRC31 through H3K9bhb. Pimozide and crizotinib targeting BDH1 can synergistically inhibit the proliferation of LUAD. This paper reveals a novel epigenetic regulatory pathway of BDH1 that regulates the malignant phenotype of LUAD, providing a new insight to treat LUAD.

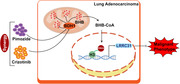

## 1 INTRODUCTION

Lung cancer is witnessing a global surge in incidence, and it currently stands as the leading cause of cancer‐related mortality.^1^ Among the various types of lung cancer, lung adenocarcinoma (LUAD) is the most prevalent, accounting for approximately 40% of all patients with lung cancer.^2^, ^3^, ^4^ In recent years, while “precision medicine” has yielded benefits for some patients with LUAD,^5^ overall cure rates for LUAD remain unsatisfactory because of the development of drug resistance during treatment.^6^, ^7^ Hence, there is an urgent need to identify potential therapeutic targets and reliable prognostic biomarkers for patients with LUAD.

Tumor metabolic reprogramming is a pivotal hallmark of cancer.^8^ Numerous genes associated with lipid metabolism have been reported to function as either oncogenes or tumor suppressor genes in LUAD.^9^ β‐Hydroxybutyrate dehydrogenase 1 (BDH1) serves as the principal rate‐limiting enzyme in ketone body metabolism, playing a key role in the interconversion of acetoacetic acid (AcAc) and β‐hydroxybutyrate (BHB) during fatty acid metabolism.^10^, ^11^ In recent years, the role of BDH1 in human tumors has garnered significant attention.^12^ BDH1 has shown abnormally higher expression in prostate cancer, acting as a potential metastasis‐associated gene in this cancer,^13^ while it has shown weak expression at lower levels in liver cancer, acute myeloid leukemia, and glioma tumors.^14^, ^15^, ^16^ The precise molecular mechanism of BDH1 in LUAD remains unclear. Elucidating the role of BDH1 in LUAD holds the promise of enhancing our comprehension of LUAD development and bears vital implications for LUAD prevention and treatment research.

Histone modifications play a crucial role in epigenetics, governing various chromatin‐related processes, including gene expression.^17^, ^18^ Recent research has revealed that BHB binds to free CoA to form BHB‐CoA, serving as a high‐energy donor for histone β‐hydroxybutyrylation (Kbhb).^19^, ^20^ Notably, H3K9bhb modification is significantly enriched at the transcription start site (TSS) of active genes that regulate gene expression and are associated with multiple metabolic pathways such as peroxisome proliferator‐activated receptor signaling pathway, fatty acid metabolism, and proteasomes.^21^ This highlights H3K9bhb modification as an important mechanism for cells to adapt to changes in cellular energy sources by regulating gene expression through epigenetic remodeling. H3K9bhb may directly link ketone body metabolism to gene regulation. In hepatocellular carcinoma, BDH1 has been shown to regulate H3K9bhb modification.^22^ Therefore, it is worth discussing whether BDH1 affects H3K9bhb modification by regulating the level of BHB in LUAD.

In recent years, targeted drugs have dominated the development of new therapeutics. However, the continued emergence of clinically acquired drug resistance often limits the sensitivity of LUAD patients to these drugs.^23^, ^24^ Hence, it is imperative to identify new indicators and strategies to combat LUAD. Small‐molecule inhibitors, which function by binding to the target protein's surface “pocket” to inhibit its function, are the primary agents used in targeted LUAD therapy.^25^ STAT3 plays a pivotal role as an oncogene in the progression of non‐small cell lung cancer (NSCLC).^26^ Zheng et al.^27^ discovered that the combination of the STAT3 inhibitor W2014‐S with gefitinib exhibited promising efficacy in NSCLC xenografts resistant to EGFR tyrosine kinase inhibitors. The activation of NRF2 is associated with lung cancer progression and chemo‐resistance.^28^ ML385, a signal transduction inhibitor of NRF2, effectively blocks the growth of NSCLC orthotopic model tumors when combined with carboplatin.^29^ Therefore, identifying oncogenes with aberrant expression in LUAD and discovering specific small molecule inhibitors are crucial for exploring innovative therapeutic approaches for LUAD.

In this study, we assessed the function of BDH1 in LUAD and revealed that BDH1 promotes the malignant phenotype of LUAD. RNA sequencing results showed that knockout of BDH1 upregulated the mRNA level of leucine‐rich repeat‐containing protein 31 (LRRC31). Our investigation confirmed that BDH1 modulates H3K9bhb modification, which mediates the regulation of the expression of LRRC31 in LUAD. Additionally, we demonstrated that pimozide and crizotinib, which are potential inhibitors of BDH1, exhibit synergistic effects and can be used in combination to target BDH1, inhibiting LUAD progression. These findings provide new insights into the malignant progression of LUAD and have important implications for LUAD prevention and treatment research.

## 2 RESULTS

### 2.1 BDH1 is strongly associated with poor prognosis in patients with LUAD

**FIGURE 1:**
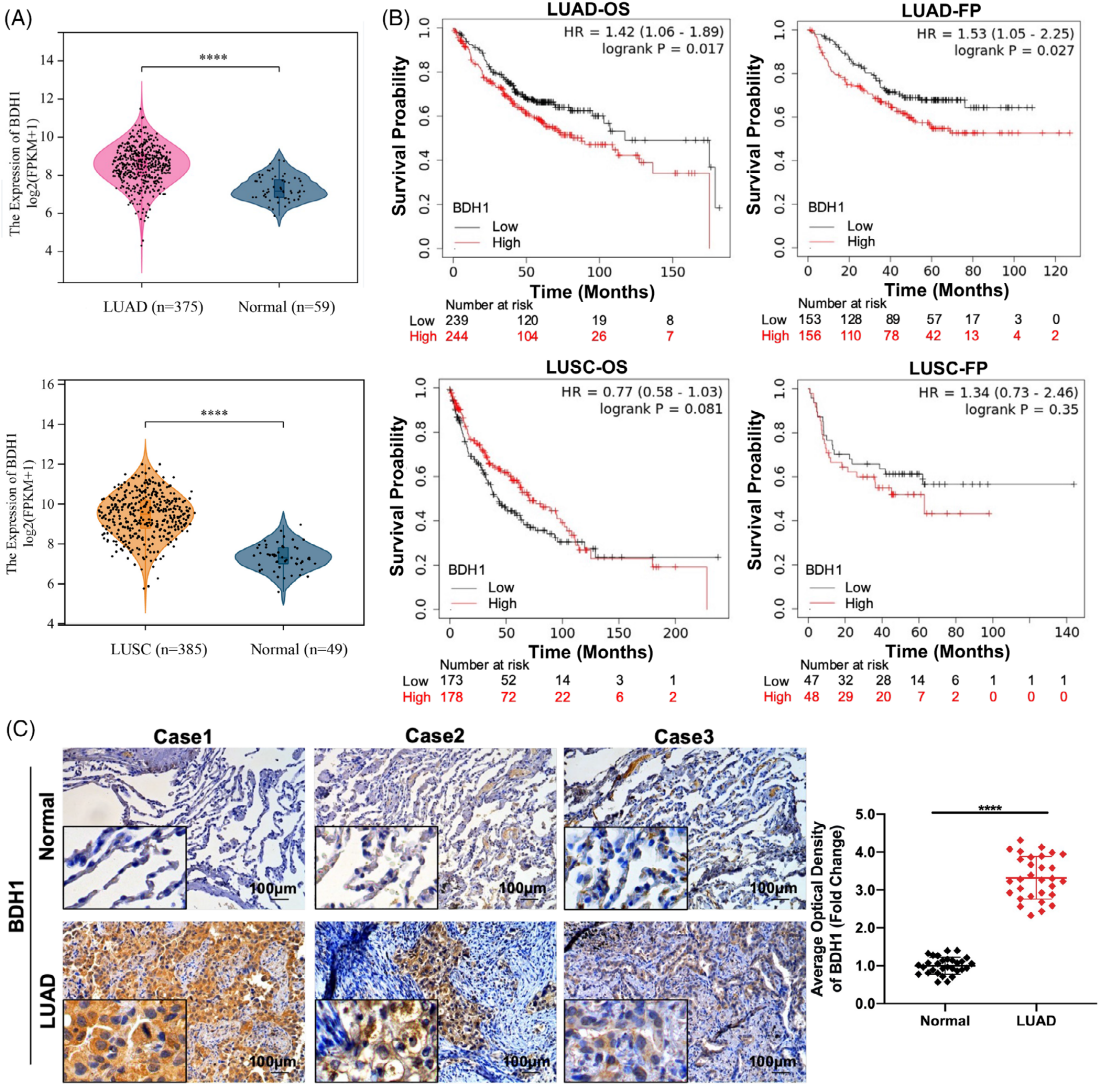
High expression of BDH1 was significantly associated with poor prognosis in LUAD. (A) The violin plot showed the expression of BDH1 in TCGA–LUAD and TCGA–LUSC. (B) Survival curves showed the correlation between the expression of BDH1 and the prognosis of patients with LUAD and LUSC. (C) IHC detected the expression of BDH1 in LUAD tissues and adjacent tissues. (****p < 0.0001; OS, overall survival; FP, first progressive).

We assessed the Cancer Genome Atlas (TCGA) dataset to characterize BDH1 expression in LUAD and lung squamous cell carcinoma (LUSC) tissues compared with adjacent normal tissues. BDH1 mRNA expression levels were significantly upregulated in TCGA–LUAD and TCGA–LUSC, relative to adjacent normal tissues (Figure 1A). The relationship between BDH1 expression levels and survival in patients with LUAD and LUSC was analyzed using Kaplan–Meier (KM) (http://kmplot.com/analysis/). The results revealed significantly lower overall survival (OS) (*p* = 0.017) and first progression (FP) (*p* = 0.027) in the high BDH1 expression group in LUAD, as opposed to the low expression group. Conversely, in LUSC, patients with lower BDH1 expression exhibited poorer OS than those with higher BDH1 expression, although this was not statistically significant (Figure 1B). Based on these findings, we chose to further investigate the expression and role of BDH1 in LUAD. To confirm BDH1 expression levels in LUAD, we assessed BDH1 protein levels in 30 pairs of human clinical LUAD and adjacent normal tissues using immunohistochemistry (IHC). This revealed a significant increase in BDH1 expression in LUAD tissues, compared with adjacent normal tissues (Figure 1C). These results suggest that the upregulated expression of BDH1 in LUAD may be carcinogenic and strongly correlated with survival in patients with LUAD.

### 2.2 BDH1 promotes the malignant phenotype of LUAD cells

**FIGURE 2:**
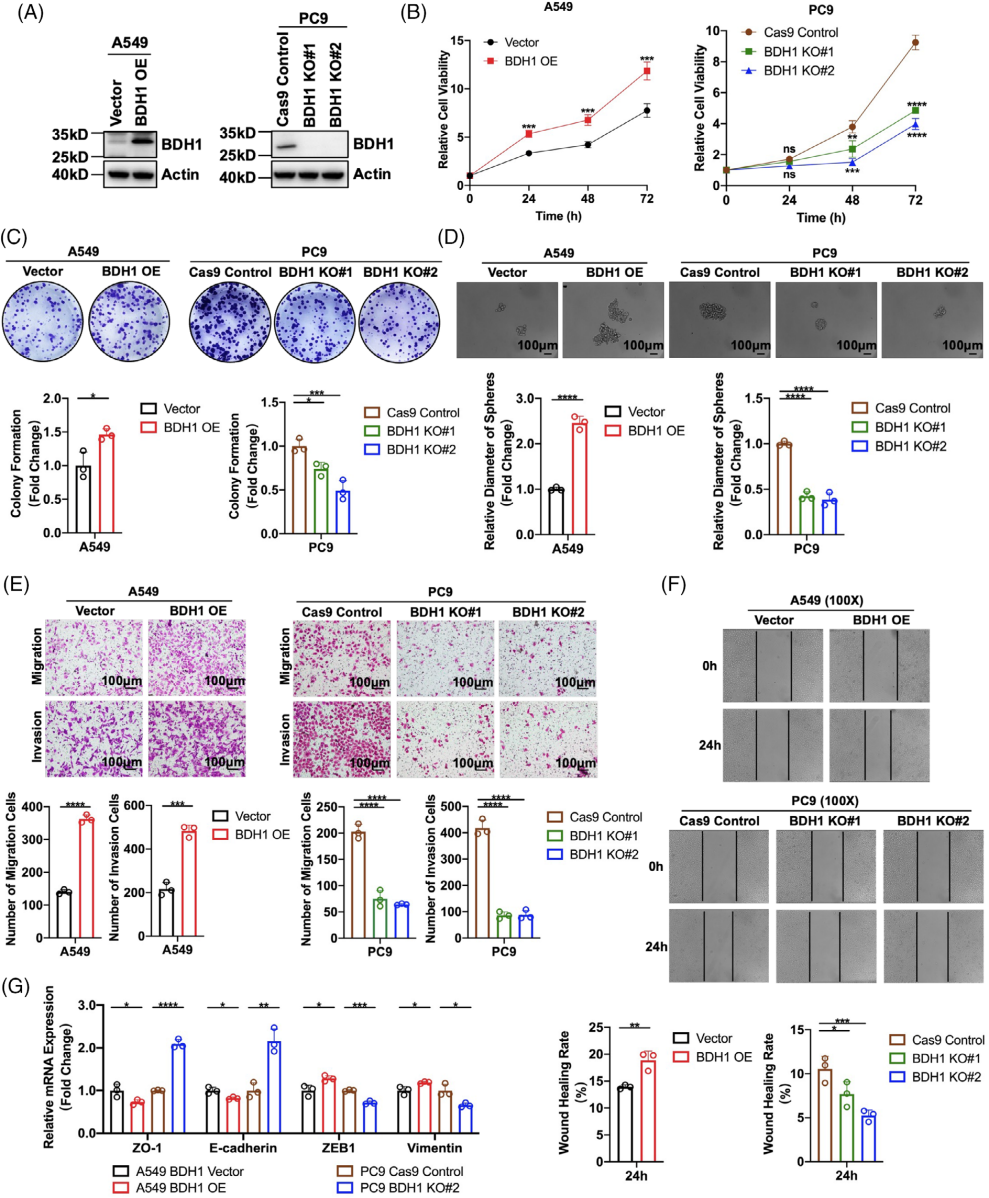
BDH1 promotes the proliferation, stem cell‐like characteristics, migration, and invasion of LUAD cells. (A) Expression levels of BDH1 in the BDH1‐overexpressing A549 cell line and BDH1‐knockout PC9 cell line were detected by WB. (B) CCK‐8 assay was used to demonstrate the proliferation ability of A549 Vector/BDH1 OE and PC9 Cas9 Control/BDH1 KO#1/BDH1 KO#2. (C) Clone formation assay was performed to detect the clone formation ability of A549 Vector/BDH1 OE and PC9 Cas9 Control/BDH1 KO#1/BDH1 KO#2. (D) Tumor‐forming assay detected stem cell‐like characteristics of A549 Vector/BDH1 OE and PC9 Cas9 Control/BDH1 KO#1/BDH1 KO#2. (E) Transwell migration and invasion assay was used to demonstrate the migration and invasion abilities of A549 Vector/BDH1 OE and PC9 Cas9 Control/BDH1 KO#1/BDH1 KO#2. (F) Wound healing assay was performed to test the wound healing ability of A549 Vector/BDH1 OE and PC9 Cas9 Control/BDH1 KO#1/BDH1 KO#2. (G) qRT‐PCR detected the expression of epithelial‐mesenchymal transition markers (ZO‐1, E‐cadherin, ZEB1, and Vimentin) in A549 Vector/BDH1 OE and PC9 Cas9 Control/BDH1 KO#2. (ns = no significant, *p < 0.05, **p < 0.01, ***p < 0.001, ****p < 0.0001).

To delve deeper into the role of BDH1 in LUAD, we selected six lung cancer cell lines and HBE cells to measure BDH1 expression levels. Our findings demonstrated high endogenous expression of BDH1 in several lung cancer cell lines (Figure S1A). Given the relatively low expression of BDH1 in A549 cells, we stably introduced exogenous overexpression of BDH1 expression in this cell. Additionally, because of higher expression levels of BDH1 in PC9 and SPCA‐1 cells, we employed three sgRNAs (Table 1) to establish stable knockout of BDH1 in these cell lines. Subsequently, western blotting (WB) confirmed a significant increase in BDH1 expression in s stable overexpression cell lines (Figure 2A), with approximately 100% knockout efficiency achieved for BDH1 KO#1 and BDH1 KO#2 in the knockout cell lines (Figures 2A and S1B). We examined the proliferative capacity of cells overexpressing or lacking BDH1 using CCK‐8 and clone formation assays. The results indicated that cell proliferation activity was substantially heightened in the BDH1 overexpression group compared with the vector control group, while it was markedly reduced in the BDH1 knockout group relative to the control group (Figures 2B–C and S1C–D). The overexpression of BDH1 led to the emergence of more stem cell‐like characteristics in the tumor sphere‐forming assay, whereas fewer stem cell‐like characteristics were observed in the BDH1 knockout group (Figures 2D and S1E).

**TABLE 1:** Targeting sgRNA to BDH1.

Name	Target sequences
BDH1 KO#1	CATAAGTCCGACGGCCAATC
BDH1 KO#2	CGGCATCTCAACGTTCGGGG
BDH1 KO#3	GATTGTCCGCTCGAGCCTGA

We conducted further assessments of cellular migration and invasion abilities using Transwell migration, Transwell invasion, and wound healing assays. These experiments revealed that BDH1‐overexpressing A549 cells exhibited heightened migration and invasion capabilities, while BDH1 knockout in PC9 and SPCA‐1 cells led to the inhibition of these processes (Figures 2E–F and S1F–G). Additionally, quantitative reverse transcription‐polymerase chain reaction (qRT‐PCR) demonstrated significantly reduced expression levels of epithelial markers (ZO‐1 and E‐cadherin) and elevated expression levels of mesenchymal markers (ZEB1 and vimentin) in the BDH1 overexpression group compared with the vector group. Conversely, the BDH1 KO#2 group showed a contrasting trend (Figures 2G and S1H). These findings collectively suggest that BDH1 promotes the malignant phenotype of LUAD cells, influencing their proliferation, stem cell‐like characteristics, migration, and invasion.

### 2.3 BDH1 inhibits the expression of LRRC31 to affect LUAD progression

To gain deeper insights into the potential molecular mechanisms of BDH1 in LUAD, we employed RNA sequencing (RNA‐seq) to analyze the downstream target genes of BDH1. The volcano plots illustrated the presentation of 313 differentially expressed genes (DEGs) in BDH1‐knockout PC9 cells versus control cells (Figure S2A; |log_2_FC| > 2, *P.adj* < 0.001). Subsequently, univariate Cox regression analysis was performed on BDH1 and the DEGs with |log_2_FC| > 4 and *P.adj* < 0.001 to identify 15 prognostic‐related genes (|HR| > 1, *p* < 0.01) (Figure S2B). Following this, we performed LASSO analysis on these 15 prognosis‐related genes. The results indicated that bias was minimized with a model containing nine genes (Figure S2C). Therefore, we selected the following candidate genes for constructing the prognostic model: BDH1, PRSS12, PTPN13, LRRC31, ETV1, PPP1R9A, CACHD1, CD33, and PABPC4L (Figure S2D). An equation was formulated to calculate the risk score for each patient: risk scores = −0.128 × ETV1 − 0.169 × CACHD1 − 0.058 × PTPN13 − 0.114 × LRRC31 − 0.012 × PRSS12 − 0.118 × CD33 − 0.323 × PABPC4L − 0.044 × PPP1R9A + 0.149 × BDH1. We employed GSE72094 (*n* = 349) as the training cohort and TCGA–LUAD (*n* = 375) as the validation cohort for the analysis of our prognostic model. Within the training cohort, patients were categorized into high‐ and low‐risk groups based on their median risk scores. We found that patients in the high‐risk group exhibited markedly lower OS than those in the low‐risk group (Figures S2E–F). The reliability of our prediction model was confirmed through risk score‐based time‐dependent receiver operating characteristics (ROC) curve analysis, which yielded area under the curve (AUC) values of 0.75, 0.75, and 0.78 at 1, 3, and 5 years, respectively (Figure S2G). These results underscore the robust predictive efficacy of our prognostic model for anticipating the survival of patients with LUAD. Furthermore, we stratified the samples into three groups based on the clinical stage of patients with LUAD (stage I, II, and III+IV) and observed that stage I exhibited the lowest risk score (Figure S2H). In the validation cohort, we observed that the results of the heat map visualization, KM curve, and clinical staging risk score distribution mirrored those of the training cohort. The AUC of the ROC curve reached 0.72, 0.69, and 0.70 at 1, 3, and 5 years, respectively, further validating the external applicability of our model (Figures S2I–L). These findings demonstrate that our model can swiftly identify high‐risk groups associated with BDH1 expression, enabling a timely assessment of the patient's future disease risk.

**FIGURE 3:**
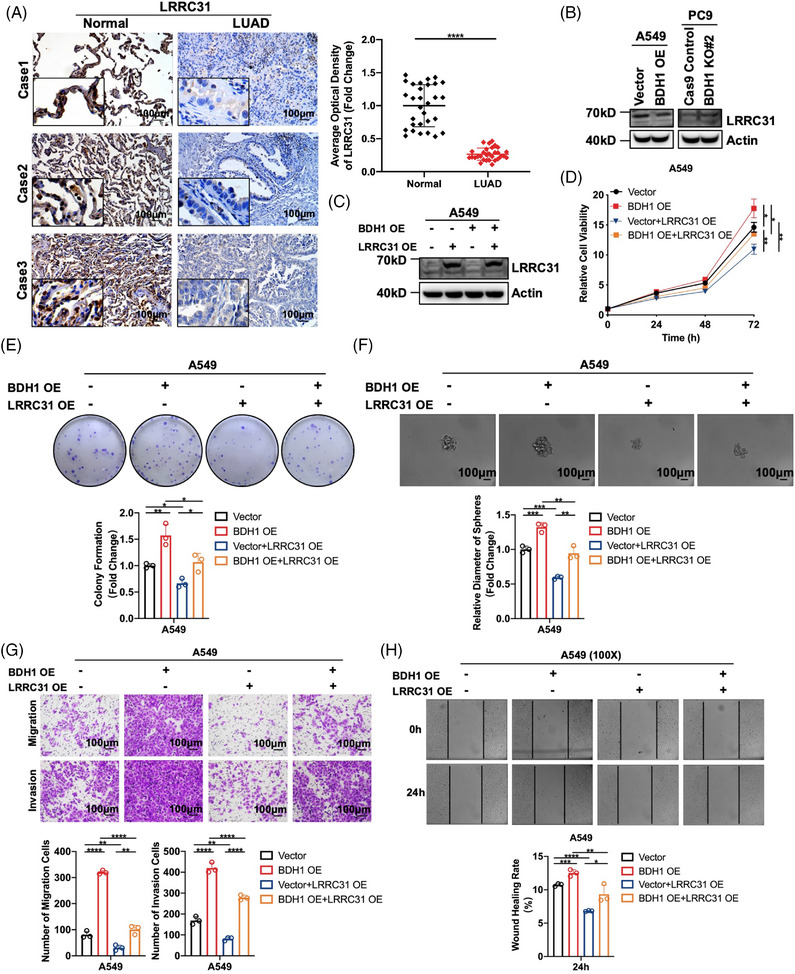
BDH1 is involved in LUAD progression by regulating LRRC31. (A) IHC detected the expression of LRRC31 in LUAD tissues and adjacent tissues. (B) Expression levels of LRRC31 in A549 Vector/BDH1 OE and PC9 Cas9 Control/BDH1 KO#2 were detected by WB. (C) The expression of LRRC31 in the A549 Vector/BDH1 OE cell line stably overexpressing LRRC31 was detected by WB. (D) CCK‐8 assay was used to demonstrate the effect of stable overexpression of LRRC31 on the proliferation of the A549 Vector/BDH1 OE cell line. (E) Clone formation assay was performed to detect the effect of stable overexpression of LRRC31 on the clonogenic ability of the A549 Vector/BDH1 OE cell line. (F) Tumor sphere formation assay detected the effect of stable overexpression of LRRC31 on the stem cell‐like characteristics of the A549 Vector/BDH1 OE cell line. (G) Transwell migration and invasion assay was used to demonstrate the effect of stable overexpression of LRRC31 on the migration and invasion of the A549 Vector/BDH1 OE cell line. (H) Wound healing assay was performed to test the effect of stable overexpression of LRRC31 on the wound healing ability of the A549 Vector/BDH1 OE cell line. (*p < 0.05, **p < 0.01, ***p < 0.001, ****p < 0.0001).

To further understand the molecular mechanisms driving BDH1‐mediated promotion of LUAD progression, we conducted qRT‐PCR analysis to evaluate the expression levels of key genes following overexpression or knockout of BDH1. The results showed that overexpression of BDH1 led to a 0.515 decrease in LRRC31 expression, while CD33, PTPN13, and CACHD1 exhibited decreases of 0.269, 0.202, and 0.172, respectively. Among the eight target genes investigated in this study for their association with BDH1 overexpression, LRRC31 displayed the most significant fold reduction in expression levels (Figure S3A). LRRC31, characterized by its nine leucine‐rich repeats, is induced by interleukin 13 to regulate esophageal epithelial barrier function in eosinophilic esophagitis.^30^ In addition, LRRC31 has been identified as a master regulator of the nonhomologous end‐joining mechanism and plays a central role in breast cancer radiotherapy.^31^ However, its expression and role in LUAD remain unclear. Patients in GSE72094 and TCGA–LUAD were stratified into high‐expression and low‐expression groups based on the median expression of LRRC31. KM survival analysis indicated that the high‐expression group exhibited markedly improved OS compared with the low‐expression group, signifying a favorable prognosis (Figure S3B). We employed qRT‐PCR and WB techniques to assess the expression of LRRC31 in various lung cancer cell lines, revealing that LRRC31 was expressed at lower levels across different lung cancer cell lines (Figures S3C–D). Additionally, IHC found lower expression of LRRC31 in LUAD tissues compared with adjacent normal tissues (Figure 3A). Subsequently, we conducted a correlation analysis of BDH1 and LRRC31 expression in LUAD tissues, revealing a negative correlation between BDH1 and LRRC31 in the LUAD tissue (*R* = 0.432, *p* = 0.018) (Figure S3E). qRT‐PCR and WB demonstrated that both the mRNA and protein expression of LRRC31 decreased in the BDH1‐overexpressing group compared with the vector cells, but increased in the BDH1 KO#2 group (Figures S3A and 3B). These findings strongly suggest that LRRC31 may function as a downstream target gene regulated by BDH1.

To elucidate the role of LRRC31 in BDH1‐mediated promotion of LUAD, we successfully achieved overexpression of LRRC31 in A549 Vector/BDH1 OE cells, while knockdown of LRRC31 was accomplished in PC9 BDH1 KO#2 cells (Figures 3C and S4A). Through a battery of assays including CCK‐8, clone formation, tumor sphere formation, Transwell migration, Transwell invasion, and wound healing, we demonstrated that overexpression of LRRC31 significantly suppressed the promotion of cell proliferation, stem cell‐like characteristics, migration, and invasion induced by BDH1 overexpression. Conversely, the knockdown of LRRC31 reversed the inhibitory effects of BDH1 knockout on these cellular processes (Figures 3D–H and S4B–F). These findings strongly suggest that LRRC31 plays a pivotal role in BDH1 regulation of LUAD.

### 2.4 BDH1 affects LUAD progression by regulating Kbhb modification

**FIGURE 4:**
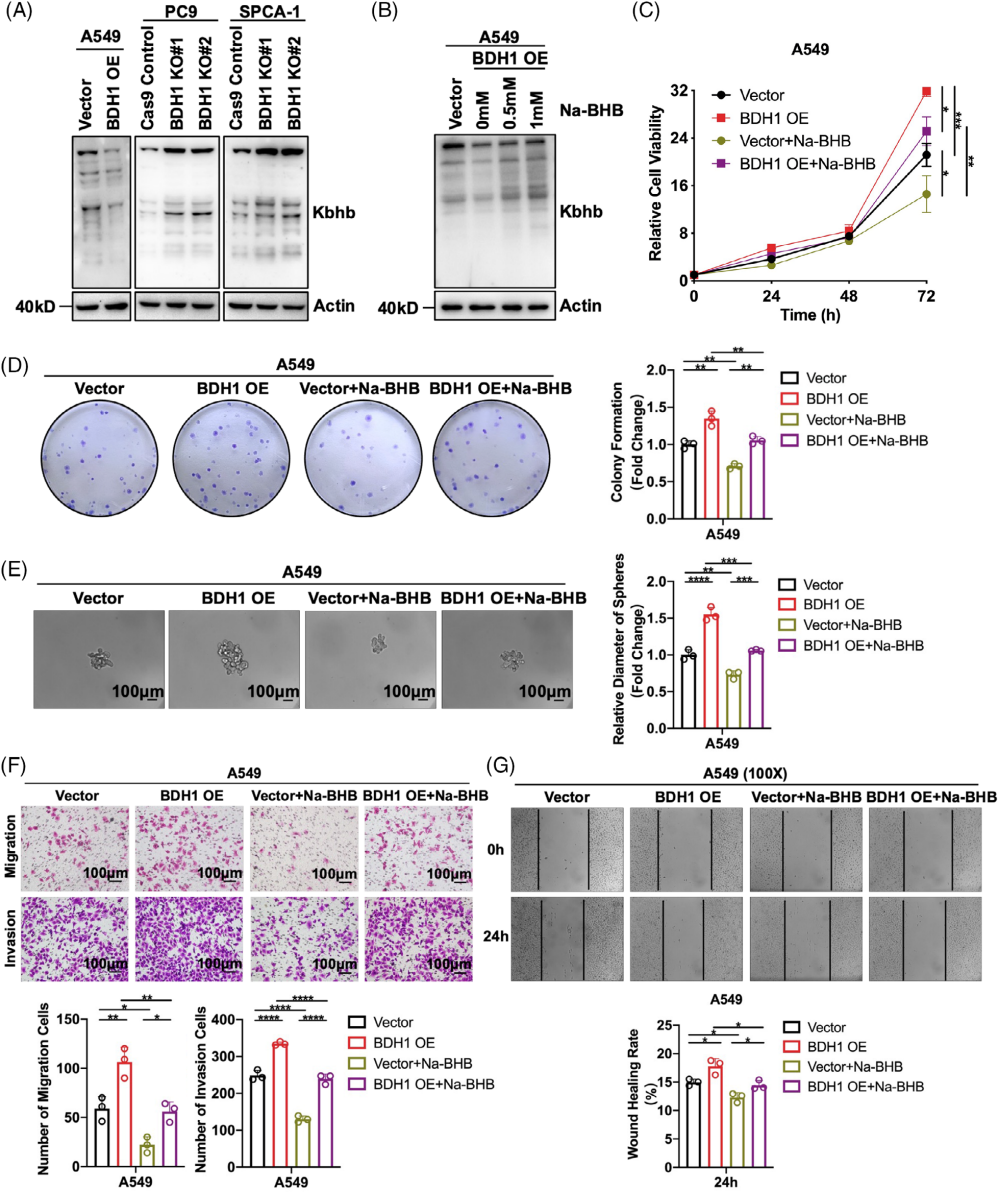
BDH1 affects LUAD progression by regulating Kbhb modification. (A) WB was performed to detect the level of Kbhb modification in A549 Vector/BDH1 OE, PC9 Cas9 Control/BDH1 KO#1/BDH1 KO#2, and SPCA‐1 Cas9 Control/BDH1 KO#1/BDH1 KO#2 cell lines. (B) Changes in the level of Kbhb modification after Na‐BHB concentration gradient treatment of A549 Vector/BDH1 OE were examined by WB. (C) CCK‐8 assay was used to demonstrate the effect of Na‐BHB on the proliferation ability of the A549 Vector/BDH1 OE cell line. (D) The effect of Na‐BHB on the clonogenic ability of the A549 Vector/BDH1 OE cell line was detected by clone formation assay. (E) Tumor sphere‐forming assay was performed to detect the effect of Na‐BHB on the stem cell‐like characteristics of the A549 Vector/BDH1 OE cell line. (F) Transwell migration and invasion assay examined the effect of Na‐BHB on the migration and invasion of the A549 Vector/BDH1 OE cell line. (G) Wound healing assay detected the effect of Na‐BHB on the wound healing ability of the A549 Vector/BDH1 OE cell line. (*p < 0.05, **p < 0.01, ***p < 0.001, ****p < 0.0001).

In ketone body metabolism, BDH1 is the primary rate‐limiting enzyme responsible for converting AcAc and BHB.^10^ Given the ability of BHB to mediate the occurrence of Kbhb,^19^ we investigated BDH1's effect on Kbhb. WB revealed that overexpression of BDH1 led to a downregulation of Kbhb levels, and vice versa (Figure 4A). Subsequently, A549 Vector/BDH1 OE cells were exposed to varying concentrations of Na‐BHB. The results demonstrated that Na‐BHB was capable of reversing the attenuating effect of BDH1 overexpression on Kbhb levels, with Kbhb levels increasing in tandem with Na‐BHB concentration (Figure 4B). These findings suggest that BDH1/BHB can influence Kbhb modification in LUAD cells. To further explore the potential effect of BHB on LUAD, we conducted a series of functional experiments. We observed that A549 cells overexpressing BDH1 exhibited a significant decrease in cell proliferation, stem cell‐like characteristics, as well as migration and invasion abilities after Na‐BHB treatment (Figures 4C–G). This indicates that Na‐BHB exerts an effective tumor‐suppressive effect. Given the reduction in Kbhb levels after BDH1 overexpression, the subsequent rise in Kbhb with the addition of Na‐BHB, and the noted tumor‐inhibitory effect of Na‐BHB, it can be inferred that BDH1 contributes to LUAD progression through Kbhb modification.

### 2.5 H3K9bhb is involved in LRRC31 regulation by BDH1 in LUAD

**FIGURE 5:**
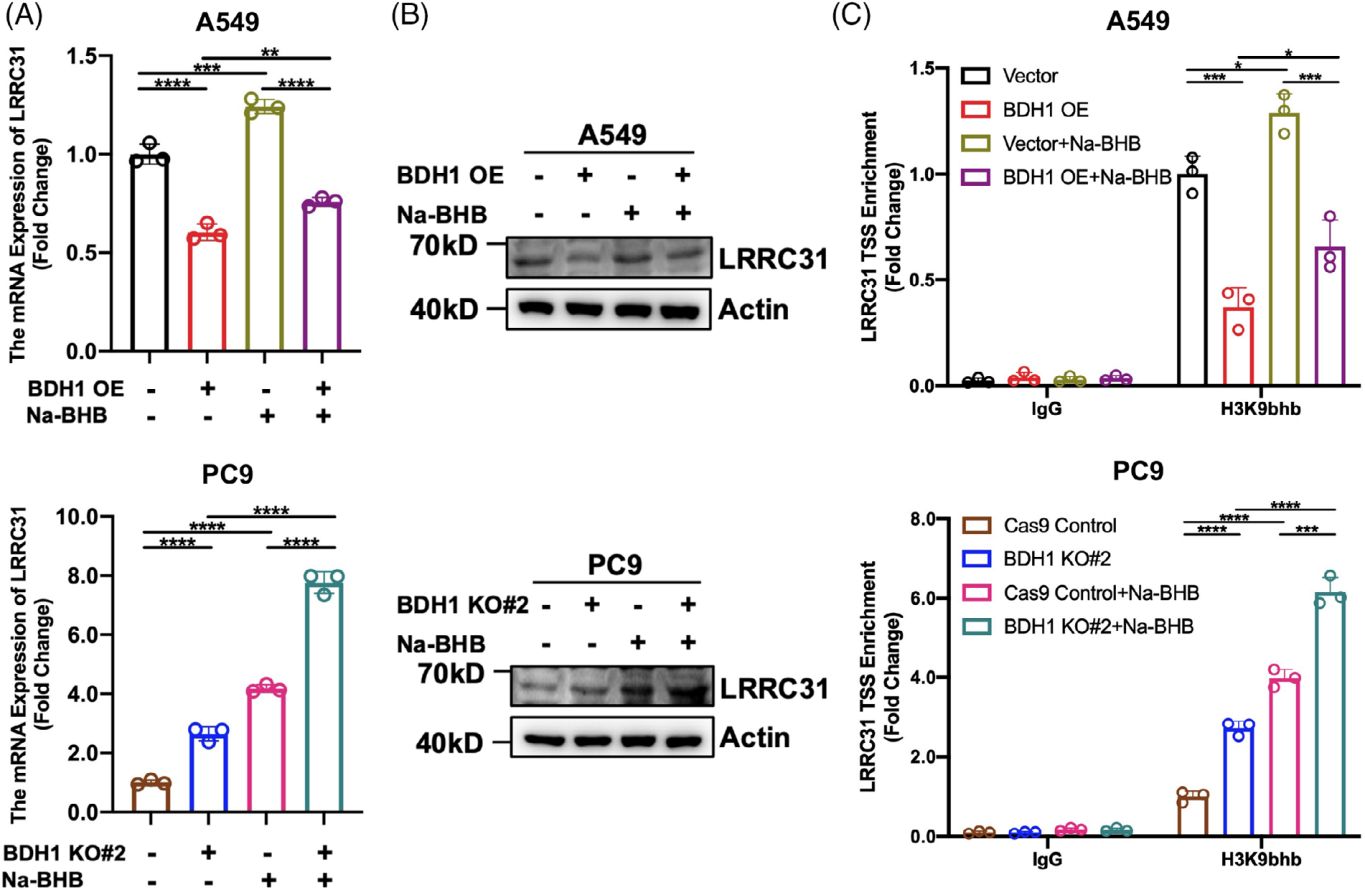
BDH1 affects the expression of LRRC31 through H3K9bhb. (A) Changes in LRRC31 mRNA levels of A549 Vector/BDH1 OE and PC9 Cas9 Control/BDH1 KO#2 after exposure to Na‐BHB were detected by qRT‐PCR. (B) WB detected changes in LRRC31 protein expression of A549 Vector/BDH1 OE and PC9 Cas9 Control/BDH1 KO#2 after exposure to Na‐BHB. (C) ChIP assay was carried out to detect the enrichment of H3K9bhb in the TSS of LRRC31 in A549 Vector/BDH1 OE and PC9 Cas9 Control/BDH1 KO#2 after exposure to Na‐BHB. (*p < 0.05, **p < 0.01, ***p < 0.001, ****p < 0.0001).

A previous study has reported that Kbhb can participate in various physiological processes, including gene expression regulation.^20^ As BDH1 can regulate the occurrence of Kbhb modifications and LRRC31 is a downstream target gene of BDH1, we sought to understand whether BDH1 mediates LRRC31 expression through Kbhb. We observed an increase in LRRC31 expression after Na‐BHB treatment of A549 Vector/BDH1 OE and PC9 Cas9 Control/BDH1 KO#2 cells (Figures 5A–B), suggesting that BDH1 regulates LRRC31 expression through Kbhb. Literature has previously reported that H3K9bhb can be enriched in the gene TSS to promote transcription.^19^ To confirm whether BDH1 affects the enrichment of H3K9bhb in the TSS of LRRC31, we performed a chromatin immunoprecipitation (ChIP) analysis. The results showed that H3K9bhb was less enriched in the TSS of LRRC31 in A549 cells overexpressing BDH1 than in vector cells, while it was more enriched in the TSS of LRRC31 in PC9 cells with BDH1 knockout compared with control cells (Figure 5C). Furthermore, the enrichment of H3K9bhb in the TSS of LRRC31 was higher after Na‐BHB treatment (Figure 5C). In conclusion, our findings demonstrate that BDH1 regulates LRRC31 expression in a H3K9bhb‐dependent manner.

### 2.6 BDH1 promotes the growth of LUAD transplantation tumors

**FIGURE 6:**
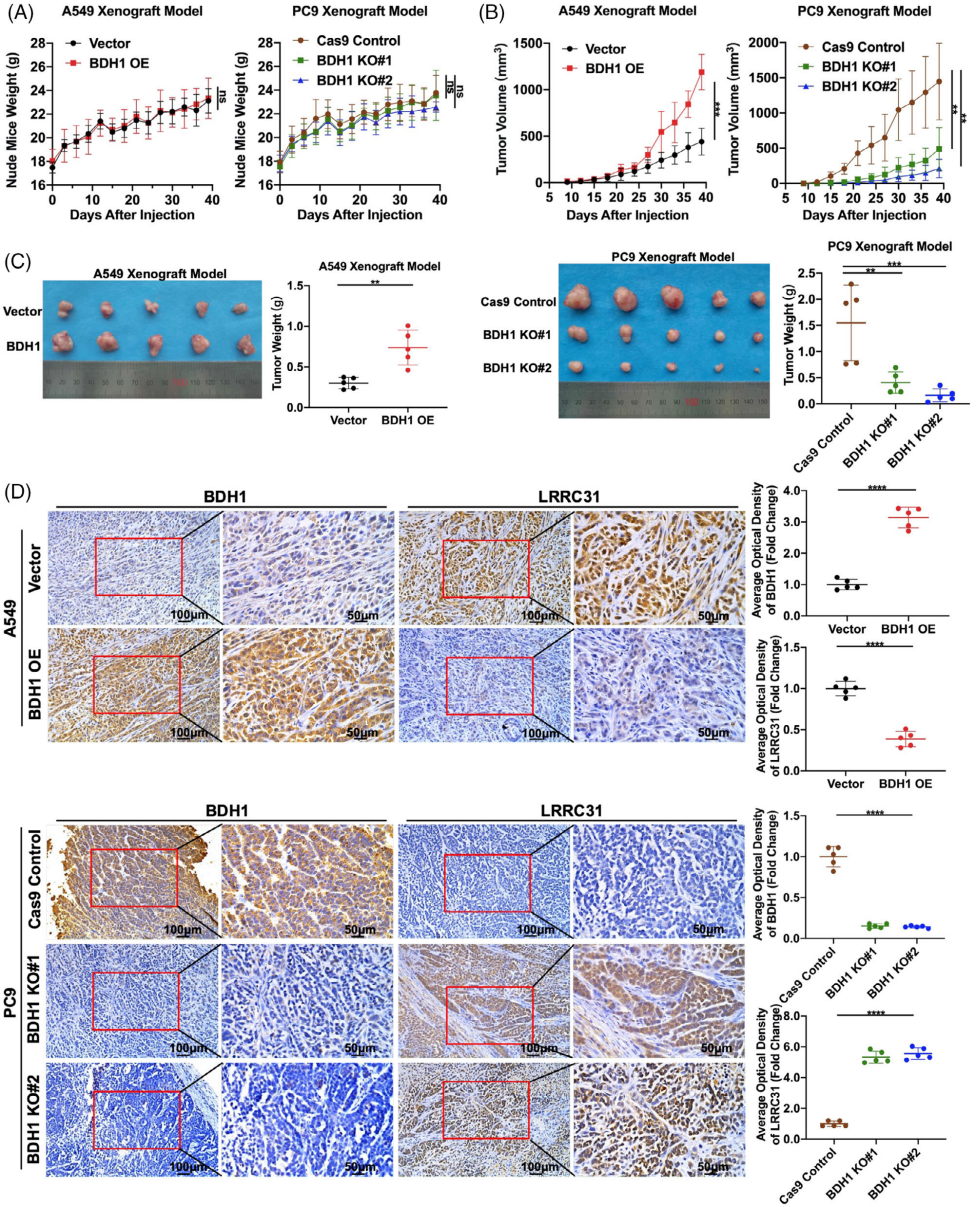
BDH1 promotes the growth of LUAD transplanted tumors. (A) Weight change of nude mice after A549 Vector/BDH1 OE and PC9 Cas9 Control/BDH1 KO#1/BDH1 KO#2 cells were injected subcutaneously in the axilla of nude mice. (B) Volume changes of transplanted tumors after A549 Vector/BDH1 OE and PC9 Cas9 Control/BDH1 KO#1/BDH1 KO#2 cells were injected subcutaneously in the axilla of nude mice. (C) A549 Vector/BDH1 OE and PC9 Cas9 Control/BDH1 KO#1/BDH1 KO#2 cells were injected subcutaneously in the axilla of nude mice, and transplanted tumors and the weight of the transplanted tumors were obtained after 39 days. (D) IHC detected the expression of BDH1 and LRRC31 in LUAD transplanted tumors. (ns = no significant, **p < 0.01, ***p < 0.001, ****p < 0.0001).

Through in vivo experiments, A549 Vector/BDH1 OE or PC9 Cas9 Control/BDH1 KO cells were subcutaneously injected into nude mice to establish xenograft models. The body weight of the nude mice was continuously monitored, and it was observed that the body weight of all five groups of nude mice steadily increased over 39 days following the injection of tumor cells, with no significant differences between the groups (Figure 6A). Compared with the vector group, the BDH1 overexpression group exhibited significantly larger tumors within the first 39 days after tumor cell injection, whereas a contrasting trend was observed in the BDH1 knockout group (Figure 6B). After 39 days of tumor cell injection, tumors from all five groups of nude mice were collected for size and weight comparison. As depicted in Figure 6C, we noted a substantial increase in both size and weight of tumors in the BDH1 overexpression group. Conversely, the tumor size and weight were significantly reduced in the BDH1 KO group. Subsequently, we conducted paraffin fixation and sectioning of the collected tumors. IHC was performed to detect the expression of BDH1 and LRRC31 in the tumors. The results revealed a significant increase in positive staining for BDH1 in the BDH1 overexpression group, along with a notable reduction in positive LRRC31 staining. In the BDH1 knockout group, BDH1 staining was predominantly negative, while positive LRRC31 staining was significantly increased (Figure 6D). This further validates the efficacy of BDH1 overexpression and knockout, as well as the suppressive influence of BDH1 on LRRC31. Collectively, the above data strongly indicate that BDH1 assumes a crucial oncogenic role in vivo, actively promoting the growth of LUAD cell xenografts.

### 2.7 Pimozide and crizotinib targeting BDH1 can synergistically inhibit the proliferation of BDH1‐high expressing LUAD cells

**FIGURE 7:**
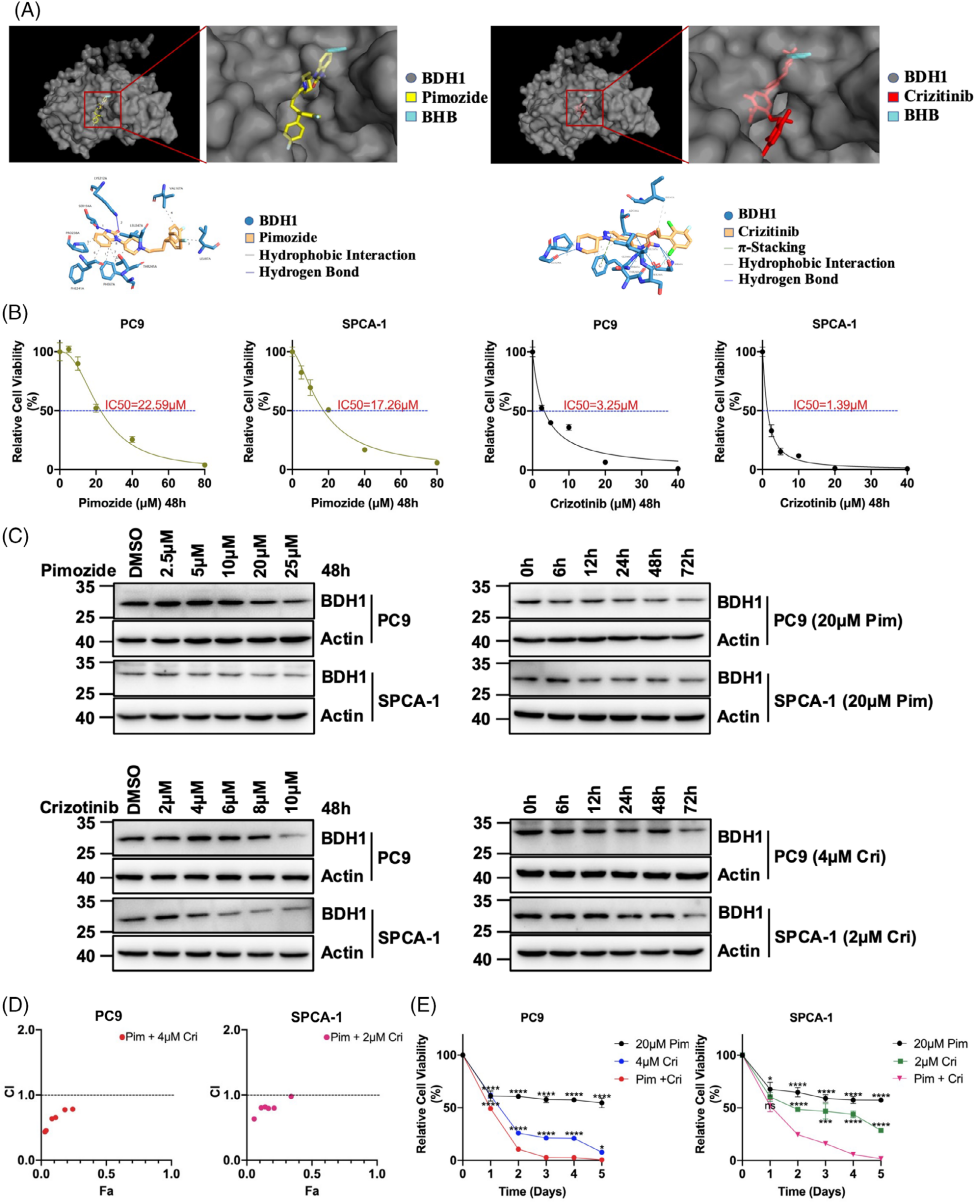
Targeting BDH1 with pimozide and crizotinib synergistically inhibited cell proliferation in LUAD. (A) Left: Visualization of the BDH1–pimozide complex and BDH1–crizotinib complex after docking. Right: Detailed view of the binding sites of BDH1–pimozide complex and BDH1–crizotinib complex. (B) Determination of IC50 of pimozide and crizotinib against PC9 and SPCA‐1. (C) BDH1 expression after pimozide or crizotinib concentration gradient and time gradient treatment of PC9 and SPCA‐1. (D) Visualization of the combination index between PC9 and SPCA‐1 treated with different concentrations of pimozide combined with crizotinib. (E) CCK‐8 assay was used to detect the survival rate of PC9 and SPCA‐1 cells treated with the combination of pimozide and crizotinib. (ns = no significant, *p < 0.05, ***p < 0.001, ****p < 0.0001; Pim, Pimozide; Cri, Crizotinib).

At present, effective inhibitors targeting BDH1 in LUAD have not been validated. To identify potential BDH1 inhibitors, we conducted a receptor‐based virtual screening utilizing AutoDock Vina, employing small molecule compounds from the ZINC database as ligands. The top 5 inhibitors with the highest docking scores were identified as pimozide, lumacaftor, nebivolol, eltrombopag, and crizotinib (Table S1). To analyze the interaction between BDH1 and the small molecule compounds with the top five docking scores, molecular dynamics (MD) simulations were performed using Gromacs. Root mean square deviation and root mean square fluctuation were computed based on the simulation trajectories. The results indicated that the carbon skeleton of BDH1, when docked with pimozide or crizotinib, exhibited acceptable fluctuations throughout the simulation, maintaining equilibrium until the end of the process. Furthermore, the small molecule ligands (pimozide or crizotinib) did not diffuse away from their initial binding sites and their conformations remained stable within 0.1 nm (Figures S5A–B), confirming the reliability of the molecular docking and MD simulation outcomes. Additionally, in both the BDH1–pimozide and BDH1–crizotinib complexes, a specific region (atoms 3500−4500) displayed increased flexibility. In both complexes, there was less fluctuation at the C‐terminus relative to the N‐terminal residues (Figures S5C–D). These findings suggest that BDH1 can form stable interactions with pimozide or crizotinib. Upon visualizing BDH1–pimozide complexes and BDH1–crizotinib complexes using Pymol, it became evident that the binding site of pimozide or crizotinib to BDH1 is adjacent to the site where the substrate BHB binds to BDH1. Additionally, the BDH1–pimozide complex forms two hydrogen bonds through active residues SER194A and LYS212A, while the BDH1–crizotinib complex forms seven hydrogen bonds involving reactive residues SER65A, PHE67A, GLY68A, ASN144A, and GLY239A (Figures 7A and Table S2–S7). These results strongly suggest that pimozide and crizotinib may serve as potential inhibitors of BDH1.

We exposed BDH1 highly expressed PC9 and SPCA‐1 cells to pimozide or crizotinib for 48 h and determined their respective IC_50_ values. The IC50 of pimozide for PC9 and SPCA‐1 was 22.59 and 17.26 μM, respectively. Crizotinib exhibited IC_50_ values of 3.25 μM for PC9 and 1.39 μM for SPCA‐1 (Figure 7B). Upon treating LUAD cells with pimozide or crizotinib, we observed a gradual downregulation of BDH1 expression with increasing treatment time or concentration, indicating a significant inhibitory effect of these drugs on BDH1 expression (Figure 7C). Furthermore, the treatment of PC9 and SPCA‐1 cells with pimozide or crizotinib for 48 h led to an upregulation of LRRC31 and H3K9bhb expression compared with the control group (Figures S6A–B). ChIP analysis unveiled a higher enrichment of H3K9bhb in the LRRC31 TSS following treatment with pimozide or crizotinib (Figure S6C), implying that these compounds exert inhibitory effects on the BDH1/H3K9bhb/LRRC31 pathway.

Crizotinib is a US FDA‐approved first‐line agent for the treatment of patients with locally advanced or metastatic NSCLC who are ALK‐positive.^32^ Therefore, we sought to determine whether pimozide could synergize with crizotinib to enhance the cytotoxic effect on LUAD cells. PC9 and SPCA‐1 cells were treated with fixed concentrations of crizotinib (PC9: 4 μM, SPCA‐1: 2 μM) in combination with different concentrations of pimozide for 48 h. Subsequently, we calculated the cell survival and combination index. The drug combination index was consistently lower than “1” at various concentrations of pimozide in combination with crizotinib, indicating a synergistic effect between crizotinib and pimozide in the treatment of LUAD cells with high expression of BDH1 (Figure 7D). We further treated PC9 with a combination of 20 μM pimozide and 4 μM crizotinib, and SPCA‐1 with a combination of 20 μM pimozide and 2 μM crizotinib. The results demonstrated a significantly reduced cell survival rate and a markedly increased proliferation inhibition rate after the combined treatment compared with single‐drug treatment (Figure 7E). In conclusion, we have identified that pimozide and crizotinib target BDH1 and work synergistically to inhibit the growth of BDH1‐high expressing LUAD cells.

## 3 DISCUSSION

During carcinogenesis, cancer cells often employ alternative energetic pathways to support their growth and metastatic requirements. In addition to uncovering anomalies in glucose, amino acid, and fatty acid metabolism, it has been observed that certain seemingly inefficient metabolites play a pivotal role in cancer cell development. Elevated acetate consumption has been linked to the proliferation of various cancer types.^33^, ^34^ Furthermore, the extracellular metabolite creatine promotes metastasis and progression^34^ of colorectal cancer. Thus, delving into potential alternative nutrient metabolic routes in tumors grants us a comprehensive insight into the diversity of cancer metabolism. Targeting pivotal genes within these metabolic pathways holds significant promise in cancer treatment. Ketone body metabolism experiences dysregulation across various cancer types and exerts a notable influence on tumorigenesis.^35^, ^36^, ^37^ Consequently, enzymes associated with ketone body metabolism that exhibit differential expression in cancer may serve as potential diagnostic, prognostic, and therapeutic targets. Among these, BDH1 stands out as the principal rate‐limiting enzyme in ketone body metabolism. In this study, we report an aberrant overexpression of BDH1 in LUAD, which fosters the malignant phenotype of this cancer. Furthermore, this research unveils, for the first time, that BDH1 modulates LRRC31 expression through the involvement of H3K9bhb in the progression of LUAD. Intriguingly, we have identified that pimozide and crizotinib, which are inhibitors targeting BDH1, exhibit a synergistic effect in inhibiting the proliferation of LUAD cells with heightened BDH1 expression (Figure S7). Our discoveries not only shed light on a hitherto overlooked tumor‐promoting role of BDH1 in LUAD but also highlight the potential of targeting BDH1 as a therapeutic approach for LUAD (Figure S8).

BDH1 serves as the terminal enzyme in hepatic ketogenesis and initiates extrahepatic ketolysis. Previous research on BDH1 has primarily focused on normal organisms or metabolic disorders. However, recent studies have unveiled its dual role in tumor development, with Huang et al.^38^ demonstrating heightened BDH1 activity in nutrient‐deficient human hepatocellular carcinoma cells, leading to the reactivation of ketolytic processes and subsequent cell proliferation. Additionally, transcriptional inactivation of BDH1 is notably correlated with poor prognosis and shorter survival in patients with acute myeloid leukemia.^15^ Notably, there is a dearth of clear studies delineating BDH1's role in LUAD. Therefore, this study aimed to elucidate the expression and function of BDH1 in LUAD. Our investigation has revealed elevated BDH1 expression in LUAD, a factor strongly associated with a bleak prognosis for patients with LUAD. BDH1 plays a pivotal role in promoting the malignant characteristics of LUAD, influencing aspects such as proliferation, stem cell‐like characteristics, migration, and invasion. Further exploration into this regulatory mechanism through RNA sequencing analysis unveiled a significant upregulation of LRRC31 mRNA after BDH1 knockout. The LRR family, which encompasses LRRC31, holds considerable sway in cell cycle regulation, gene expression, apoptosis, and DNA repair.^39^ LRRC31, which is highly homologous to RNase/angiopoietin inhibitor 1, has been identified by Chen et al.^31^ as a DNA repair inhibitor that interacts with Ku70/Ku80 and ATR at the protein level to impede breast cancer progression. To delve deeper into the role of LRRC31 on the malignant phenotype of LUAD influenced by BDH1, we conducted rescue experiments. These experiments demonstrated that overexpression of LRRC31 can counteract the pro‐LUAD progression induced by BDH1 overexpression. Conversely, the knockdown of LRRC31 resulted in intensified cell proliferation, stem cell‐like characteristics, migration, and invasion in BDH1 knockout cells. These findings collectively indicate that BDH1 facilitates the progression of LUAD via LRRC31.

Ketone bodies comprise a group of water‐soluble molecules featuring ketone groups, including AcAc, BHB, and acetone. Notably, BHB constitutes the most prevalent form, accounting for approximately 70% of circulating ketone bodies.^40^ BHB and AcAc serve as crucial alternate energy sources for extrahepatic tissues in conditions of glucose scarcity and various physiological states.^41^ Following their entry into circulation and absorption by extrahepatic tissues, BHB undergoes conversion to AcAc facilitated by BDH1. This initiates a sequence of reactions culminating in the generation of acetyl‐CoA into citric acid, thereby producing ATP through the tricarboxylic acid cycle and the electron transport chain.^40^ Apart from acting as an energy source for nonhepatic tissues, BHB also drives post‐translational modification. BHB forms BHB‐CoA when it binds with free CoA, serving as a high‐energy contributor for Kbhb.^21^ Histone Kbhb actively participates in the transcriptional control of genes, influencing the vital functions of organisms by reconfiguring the epigenetic profile of histones and regulating gene expression. For instance, histone Kbhb upregulates the expression of vascular endothelial growth factor to counteract aortic endothelial cell damage in cases of diabetes.^42^ BHB's antidepressant action hinges on the activation of brain‐derived neurotrophic factor by H3K9bhb.^43^ Furthermore, BHB induces the upregulation of FOXO1 and PGC1α expression through H3K9bhb, subsequently upregulating Pck1 to govern CD8^+^ memory T cell formation and maintenance.^44^ BHB is regulated by BDH1 in ketone body metabolism.^40^ This connection potentially links ketone body metabolism, Kbhb, and gene expression control. Inhibition of BDH1, as previously reported, results in increased levels of H3K9bhb, consequently upregulating the expression of multiple genes and fostering the proliferation of hepatocellular carcinoma cells.^22^ Hence, our research hones in on the influence of H3K9bhb, under the aegis of BDH1, in steering LUAD development. Indeed, our study underscores that BDH1 exerts an influence on the modification of Kbhb, contributing to LUAD progression, and this process hinges on H3K9bhb in LUAD to regulate the expression of LRRC31.

Small‐molecule inhibitors play a crucial role in combining protein targets to control the proliferation, survival, apoptosis, and differentiation of malignant tumors, offering convenience and effectiveness, particularly for patients with specific genetic alterations or distinct characteristics. Notably, mutations in EGFR tyrosine kinases are pivotal in NSCLC progression,^45^ establishing inhibitors targeting EGFR tyrosine kinase as the primary treatment for NSCLC carrying EGFR exon 19 deletion or exon 21 mutation.^25^ Nevertheless, the emergence of acquired drug resistance has diminished the efficacy of numerous drugs,^46^ underscoring the pressing need for alternative dosing regimens in these cases. Previously, BDH1's significant role in promoting LUAD progression was established. Our research pioneered the identification of pimozide and crizotinib as potential BDH1 inhibitors, effectively restraining LUAD cell proliferation through targeted interference with the BDH1/H3K9bhb/LRRC31 pathway. Studies have shown pimozide's effectiveness in inhibiting epithelial‐mesenchymal transition and migration invasion in prostate cancer and hepatocellular carcinoma by downregulating N‐cadherin expression and upregulating E‐cadherin expression.^47^, ^48^ Pimozide can also synergize with chemotherapy drugs in leukemia and ovarian cancer to increase chemotherapy effectiveness,^49^, ^50^ suggesting that combination treatment with pimozide and chemotherapeutic drugs may be a promising therapeutic approach. Crizotinib is an ATP‐competitive multi‐target protein kinase inhibitor that inhibits Met/ALK/ROS.^51^ Crizotinib capsules have been used clinically in the treatment of patients with ALK‐positive locally advanced or metastatic NSCLC. Our investigation examined the combined effect of pimozide and crizotinib on BDH1‐high expressing LUAD cells, revealing a synergistic inhibition of cell growth. Compared with single‐drug treatment, the cell growth of LUAD was more significantly inhibited when pimozide was combined with crizotinib. Therefore, the discovery of specific small‐molecule inhibitors targeting BDH1 holds promise for the development of novel treatments for LUAD.

Nonetheless, this study has several limitations. First, it does not address the impact of potential BDH1 inhibitors on LUAD migration, invasion, and tumor transplantation. Second, exploring the synergistic effects of pimozide with other LUAD chemotherapy drugs in inhibiting disease progression warrants further investigation. These avenues will be pursued in future studies, and we encourage collaboration with scholars in related fields to conduct pertinent experiments, thus advancing our understanding in this domain.

## 4 CONCLUSION

This study unveils the epigenetic regulatory pathway of BDH1 in governing LUAD development, offering the first elucidation of the molecular mechanism involving the BDH1/H3K9bhb/LRRC31 axis in mediating LUAD progression. Additionally, it clarifies the synergistic effect of pimozide and crizotinib, specific BDH1‐targeting inhibitors, in inhibiting the proliferation of BDH1‐high‐expressing LUAD cells. These findings position BDH1 as a potential therapeutic target for LUAD, providing a robust theoretical and experimental foundation for drug development in this context.

## 5 MATERIALS AND METHODS

### 5.1 Data collection

Gene expression data along with corresponding clinical information for LUAD and LUSC were obtained from the TCGA database (https://portal.gdc.cancer.gov). RNA‐seq data and clinical follow‐up details for patients with LUAD in the GSE72094 dataset were accessed from the Gene Expression Omnibus (GEO, http://www.ncbi.nlm.nih.gov/geo). After excluding patients with one or more unavailable clinical characteristics, all datasets were included in the subsequent analyses (Table S8).

### 5.2 IHC analysis

Biopsies of 30 pairs of LUAD tissues and adjacent normal tissues were provided and verified by the Department of Pathology at Xiangya Hospital of Central South University (Table S8). IHC was performed following a previously described method.^52^ The antibodies used were BDH1 (67448‐1‐Ig; Proteintech), LRRC31 (CSB‐PA744274LA01HU; CUSABIO), and horseradish peroxidase (HRP)‐labeled secondary antibodies (mouse: G1214, rabbit: G1213; Servicebio). Subsequently, images were captured using a ZEISS microscope (Germany) and analyzed by determining the average optical density of the slices using ImagePro Plus 6.0.

### 5.3 Cell culture, plasmids, and sgRNA

Human embryonic kidney 293T cells (American Type Culture Collection [ATCC]: CRL‐3216) and lung cancer cell lines A549 (ATCC: CCL‐185), H358 (ATCC: CRL‐5807), HCC827 (ATCC: CRL‐2868), and H520 (ATCC: HTB‐182) were obtained from ATCC. HBE cells and lung cancer cell lines PC9 and SPCA‐1 were acquired from the Institute of Oncology at Central South University. All cells were cultured in a medium supplemented with 10% fetal bovine serum, with specific mediums used for different cell lines. DMEM/F12 (1:1) (Gibco) was utilized for A549 cells, DMEM (Gibco) was used for 293T and HBE cell lines, and RPMI 1640 (Gibco) was used for H358, HCC827, H520, PC9, and SPCA‐1 cell lines. All cells were maintained in a cell culture incubator at 37°C with 5% CO_2._

The BDH1 overexpression plasmid, sourced from WZ Biosciences (China), was constructed by inserting BDH1 cDNA into the pCDH‐CMV‐MCS‐EF1‐Puro vector. The sgRNA vector targeting BDH1 and the corresponding control were procured from General Biol (China). The specific sgRNA oligo targeting BDH1 used in this study is outlined in Table 1.

Similarly, the LRRC31 overexpression plasmid was obtained from the MiaoLing Plasmid Platform (China) and was generated by inserting LRRC31 cDNA into the pLV3‐CMV‐EF1‐Neo vector. The shRNA vector targeting LRRC31 and the control were purchased from GeneChem (China). The shRNA sequences targeting LRRC31 used in this research are detailed in Table S9. Plasmid transfection was executed employing ExFect transfection reagent (Vazyme, China) by the manufacturer's instructions. Viral solutions were collected for cell infection, followed by screening for stable expression colonies using puromycin or G418.

### 5.4 Quantitative reverse transcription‐polymerase chain reaction

The experimental protocol was conducted as previously described,^53^ and the primers for this experiment were sourced from Biotech. The sequences for all primers are indicated in Table S10.

### 5.5 Western blotting

WB was carried out following established procedures.^54^ The following antibodies were utilized: β‐actin antibody (AF7018; Affinity), BDH1 antibody (67448‐1‐Ig; Proteintech), LRRC31 antibody (CSB‐PA744274LA01HU; CUSABIO), Kbhb antibody (PTM‐1201; PTMBIO), H3 antibody (17168‐1‐AP; Proteintech), Tubulin antibody (AF7010; Affinity), H3K9bhb antibody (PTM‐1250; PTMBIO), goat antirabbit IgG (H+L) HRP (S0001; Affinity), and goat antimouse IgG (H+L) HRP (S0002; Affinity).

### 5.6 CCK‐8 assay, colony formation assay, tumor sphere formation assay, wound healing assay, Transwell migration, and invasion assay

These methodologies have been comprehensively detailed in previous studies, and in this study, they were performed in line with established protocols.^53^, ^54^, ^55^, ^56^

### 5.7 RNA sequencing

Total RNA from PC9 BDH1 KO#2 and control cells was collected and subjected to RNA sequencing, which was performed by Majorbio Biopharmaceutical Technology (China).^53^

### 5.8 Prognostic model construction and validation

Screening for DEGs between the PC9 BDH1 KO#2 cells and the control was performed using the R package “DEGseq” (|log_2_FC| > 2, *P.adj* < 0.001). Subsequently, the R package “pheatmap” was utilized to generate a volcano plot for visualizing the results of the DEGs. Furthermore, univariate Cox regression analysis of the DEGs was conducted to evaluate their predictive significance for patient OS. Candidate genes were selected through LASSO analysis for constructing a prognostic risk model. Risk scores for each sample were computed using the following formula: risk scores = sum (expression of each candidate gene × corresponding LASSO regression coefficient).^53^ The GSE72094 dataset served as the training cohort, while TCGA–LUAD was employed as the validation cohort for analyzing the prognostic model. Prognostic heat maps were generated using the R packages “ggrisk” and “pheatmap” to illustrate the relationship between the prognostic model and the gene expression within the model. Subsequently, KM survival curves were constructed using the R package “survival”. The “pROC” R package was employed to carry out time‐dependent ROC analysis and compute the AUC values for 1, 3, and 5 years. Additionally, the “ggplot2” R package was utilized to present the risk scores of samples grouped by clinical stage in a violin plot.

### 5.9 Chromatin immunoprecipitation

ChIP experiments were conducted essentially as previously described.^52^ Antibody–protein complexes were captured using pre‐blocked protein G magnetic beads (10004D, Thermo Fisher Scientific, USA) and subsequently immunoprecipitated with either IgG (AC005; Abclonal) or H3K9bhb (PTM‐1250; PTMBIO). The primer sequences employed in this assay are listed in Table S10.

### 5.10 Nude mice xenograft lung tumor model

Four‐week‐old female BALB/c nude mice were procured from Hunan SJA Laboratory Animal Company (China). Animal experiments were conducted with the approval of the Biomedical Research Ethics Committee of Hunan Normal University. Subsequently, BDH1‐overexpressing A549 or BDH1‐knockout PC9 cells, along with their corresponding control cells, were subcutaneously injected into the axilla of each nude mice (2 × 106 cells per nude mice). Thereafter, the tumor volume and body weight of the nude mice were measured every 3 days. After stripping the tumors and recording their weights, the tumor tissues were fixed and subjected to IHC staining.

### 5.11 Virtual screening and MD

The crystal structures of BDH1 were retrieved from the UniProt database (https://www.uniprot.org), while the small molecule compounds were obtained from the ZINC database (https://zinc15.docking.org/). AutoDock Vina was employed to add hydrogen atoms to both BDH1 and the small molecule compounds. This process optimized hydrogen bonding and minimized binding energy.^57^ Subsequently, MD simulations of the BDH1–compound complexes were performed using Gromacs.^58^ Considering the balance between simulation accuracy and computational resources, MD simulations were conducted for a duration of 50 ns. Finally, the complexes of BDH1 with the compounds were visualized using Pymol.^59^

### 5.12 Statistical analysis

The experiments were replicated at least three times, except for those involving nude mice. Data are presented as mean ± standard deviation. Student's *t*‐test was applied for comparisons between groups. Data analysis was performed using R (version 4.2.0) or GraphPad Prism 9.0, and *p* values less than 0.05 were deemed statistically significant (*p < 0.05, **p < 0.01, ***p < 0.001, ****p < 0.0001).

## AUTHOR CONTRIBUTION

Y. J. and L. C. contributed to the conception and design of the article. J. H. and L. L. wrote the manuscript and contributed equally. S. J., H. H., and Y. L. (Yueying Liu) collected the references and participated in the discussion. L. X., Y. L. (Yi Li), and X. S. prepared the figures and tables. Y. T. examined the data of the article. All authors read and approved the final manuscript.

## CONFLICT OF INTERESTS STATMENT

The authors declare no conflict of interest.

## ETHICS STATEMENT

The animal experiments were conducted with the approval of the Biomedical Research Ethics Committee of Hunan Normal University (No. 439 in 2022).

## Supporting information

Supporting Information

Supporting Information

## Data Availability

The data that support the findings of this study are available from the corresponding author upon reasonable request.
