# Supplementary material for: BDH1‐mediated LRRC31 regulation dependent on histone lysine β‐hydroxybutyrylation to promote lung adenocarcinoma progression
Source: MedComm (2020). 2023 Dec 13;4(6):e449. doi: 10.1002/mco2.449 (PMC10719427; doi:10.1002/mco2.449)
Supplement: Supplementary file 2 — Supporting Information [file MCO2-4-e449-s002.pdf]

BDH1-Mediated LRRC31 Regulation Dependent on Histone Lysine β-Hydroxybutyrylation to Promote Lung Adenocarcinoma Progression

Jingjing Huang[#1,2], Lu Liang[#1,2], Shiyao Jiang[1,2], Yueying Liu[1,2], Hua He[1,2], Xiaoyan Sun[1,2], Yi Li[1,2], Li Xie[3], Yongguang Tao[4], Li Cong[†1,2], Yiqun Jiang[†,1,2]

Author Information

1 The Key Laboratory of Model Animal and Stem Cell Biology in Hunan Province, Hunan Normal University, Changsha, 410013 Hunan, China.

2 School of Medicine, Hunan Normal University, Changsha, 410013 Hunan, China.

3 Department of Head and Neck Surgery, Hunan Cancer Hospital, Xiangya School of Medicine, Central South University, Changsha, 410013 Hunan, China.

4 Key Laboratory of Carcinogenesis and Cancer Invasion, Ministry of Education, Department of Pathology, Xiangya Hospital, School of Basic Medicine, Central South University, Changsha, 410078 Hunan, China.

† Corresponding author:

Li Cong (congli@hunnu.edu.cn)

Yiqun Jiang (jiangyiqun@hunnu.edu.cn)

Yiqun Jiang, Ph.D

School of Medicine

Hunan Normal University

371 Tongzipo Road

Changsha, Hunan, 410013

China

Tel: +86-18674896369

Email: jiangyiqun@hunnu.edu.cn

Table S1 Top 5 small molecule inhibitors with BDH1 docking score

Ligand Name (Zinc)	Name	Scores
ZINC000004175630	Pimozide	-11.1
ZINC000064033452	Lumacaftor	-10.9
ZINC000001999441	Nebivolol	-10.7
ZINC000011679756	Eltrombopag	-10.6
ZINC000035902489	Crizotinib	-10.5

Table S2 Amino acid residues forming hydrophobic interactions in BDH1-pimozide complexes

Index	Residue	AA	Distance	Ligand Atom	Protein Atom
1	67A	PHE	3.14	2692	500
2	67A	PHE	3.56	2716	498
3	87A	LEU	3.36	2711	648
4	167A	VAL	3.68	2713	1268
5	238A	PRO	3.95	2689	1823
6	241A	PHE	3.97	2691	1842
7	245A	THR	3.46	2692	1872
8	247A	LEU	3.39	2692	1886

PHE: Phenylalanine; LEU: Leucine; VAL: Valine; PRO: Proline; THR: Threonine.

Table S3 Amino acid residues forming hydrogen bonds in BDH1-pimozide complexes

Index	Residue	AA	Distance H-A	Distance D-A	Donor Angle	Protein donor?	Side chain	Donor Atom	Acceptor Atom
1	194A	SER	2.08	2.87	137.00			2687 [Nam]	1484 [O2]
2	212A	LYS	2.12	2.99	141.18			1622 [N3+]	2685 [O2]

SER: Serine; LYS: Lysine.

Table S4 Amino acid residues forming hydrophobic interactions in BDH1-Crizotinib complexes

Index	Residue	AA	Distance	Ligand Atom	Protein Atom
**1**	67A	PHE	3.37	2699	500
**2**	147A	ILE	3.99	2685	1109
**3**	238A	PRO	3.93	2695	1823
**4**	245A	THR	3.47	2699	1872
**5**	247A	LEU	3.44	2699	1886

PHE: Phenylalanine; ILE: Isoleucine; PRO: Proline; THR: Threonine; ILE: Leucine.

Table S5 Amino acid residues forming hydrogen bonds in BDH1-Crizotinib complexes

Index	Residue	AA	Distance H-A	Distance D-A	Donor Angle	Protein donor?	Side chain	Donor Atom	Acceptor Atom
1	65A	SER	2.16	2.97	140.17	×	×	2703 [Npl]	484 [O2]
2	65A	SER	2.78	3.49	129.11	√	×	481 [Nam]	2705 [Npl]
3	65A	SER	2.20	2.99	138.02	×	√	2705 [Npl]	486 [O3]
4	67A	PHE	3.09	3.59	113.34	√	×	491 [Nam]	2703 [Npl]
5	68A	GLY	2.88	3.86	174.41	√	×	502 [Nam]	2703 [Npl]
6	144A	ASN	2.65	3.60	162.28	√	√	1092 [Nam]	2703 [Npl]
7	239A	GLY	1.95	2.90	156.63	×	×	2697 [N3]	1829 [O2]

SER: Serine; PHE: Phenylalanine; GLY: Glycine; ASN: Asparagine.

Table S6 Amino acid residues forming π-Stacking in BDH1-Crizotinib complexes

Index	Residue	AA	Distance	Angle	Offset	Stacking Type	Ligand Atoms
1	67A	PHE	4.79	70.67	0.48	T	2691, 2692, 2693, 2700, 2701

PHE: Phenylalanine.

Table S7 Amino acid residues forming halogen bonds in BDH1-Crizotinib complexes

Index	Residue	AA	Distance	Donor Angle	Acceptor Angle	Donor Atom	Acceptor Atom
1	246A	SER	3.39	159.68	106.05	2712 [Cl]	1877 [O2]

SER: Serine.

Table S9 Targeting shRNA to LRRC31

shRNA	Sequences
shLRRC31#1	AGCTAATCGAGCTGGATATTA
shLRRC31#2	CAGTATTGCTCAGGGATTAAA
shLRRC31#3	GCTGTCAACAAGTGTCTAGAT

Table S10 Primers for qPCR

Primers	Forward	Reverse
BDH1	GAAAGTGGTGGAGATTGTCCGC	TGTAGGTCTCCAGGCTGGTGAA
LRRC31	GCTCACTAACAGCAGATGACGTG	GGTAAAAATCGGAGCCTGCTGAG
Vimentin	AGGCAAAGCAGGAGTCCACTGA	ATCTGGCGTTCCAGGGACTCAT
N-Cadherin	CCTCCAGAGTTTACTGCCATGAC	GTAGGATCTCCGCCACTGATTC
ZEB1	GGCATACACCTACTCAACTACGG	TGGGCGGTGTAGAATCAGAGTC
β-Catenin	CACAAGCAGAGTGCTGAAGGTG	GATTCCTGAGAGTCCAAAGACAG
ZO-1	GTCCAGAATCTCGGAAAAGTGCC	CTTTCAGCGCACCATACCAACC
Snail	TGCCCTCAAGATGCACATCCGA	GGGACAGGAGAAGGGCTTCTC
Slug	ATCTGCGGCAAGGCGTTTTCCA	GAGCCCTCAGATTTGACCTGTC
E-Cadherin	GCCTCCTGAAAAGAGAGTGGAAG	TGGCAGTGTCTCTCCAAATCCG
Actin	CACCATTGGCAATGAGCGGTTC	AGGTCTTTGCGGATGTCCACGT

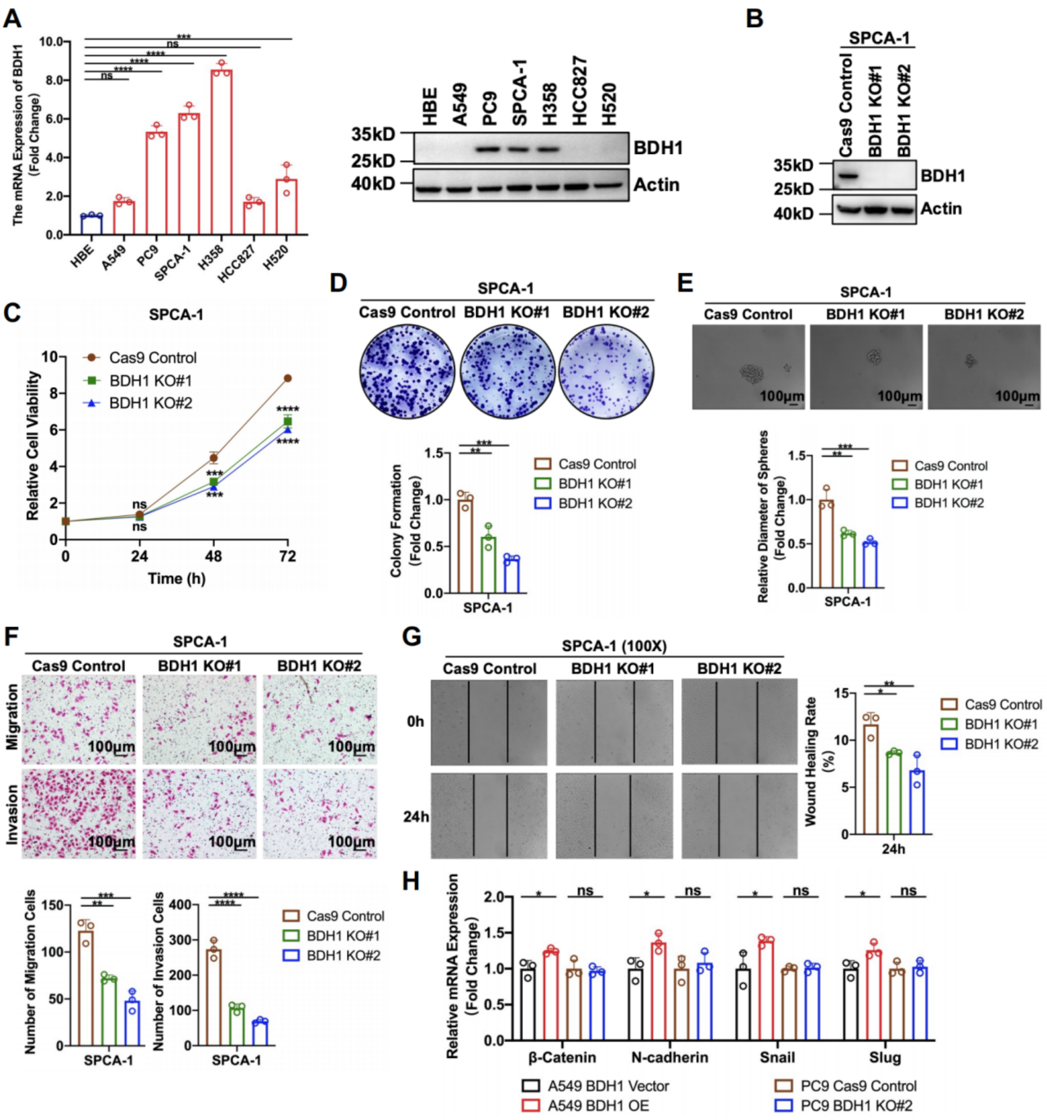

Figure S1 Knockout of BDH1 inhibits the proliferation, stem cell-like characteristics, migration, and invasion of LUAD cells. (A) qRT-PCR and WB examined the expression level of BDH1 in lung cancer cells and normal cells.（B） Expression levels of BDH1 in the BDH1-knockout SPCA-1 cell line were detected by WB. (C) CCK-8 assay was used to demonstrate the proliferation ability of SPAC-1 Cas9 Control/BDH1 KO#1/BDH1 KO#2. (D) Clone formation assay was performed to detect the clone formation ability of SPAC-1 Cas9 Control/BDH1 KO#1/BDH1 KO#2. (E) Tumor-forming assay detected stem cell-like characteristics of SPAC-1 Cas9 Control/BDH1 KO#1/BDH1 KO#2. (F) Transwell migration and invasion assay was used to

demonstrate the migration and invasion abilities of SPAC-1 Cas9 Control/BDH1 KO#1/BDH1 KO#2. (G) Wound healing assay was performed to test the wound healing ability of SPAC-1 Cas9 Control/BDH1 KO#1/BDH1 KO#2. (H) qRT-PCR detected the expression of epithelial-mesenchymal transition markers (β-catenin, N-cadherin, Snail, and Slug) in A549 Vector/BDH1 and PC9 Cas9 Control/BDH1 KO#2. (ns = no significant, * $P < 0.05$, ** $P < 0.01$, *** $P < 0.001$, **** $P < 0.0001$)

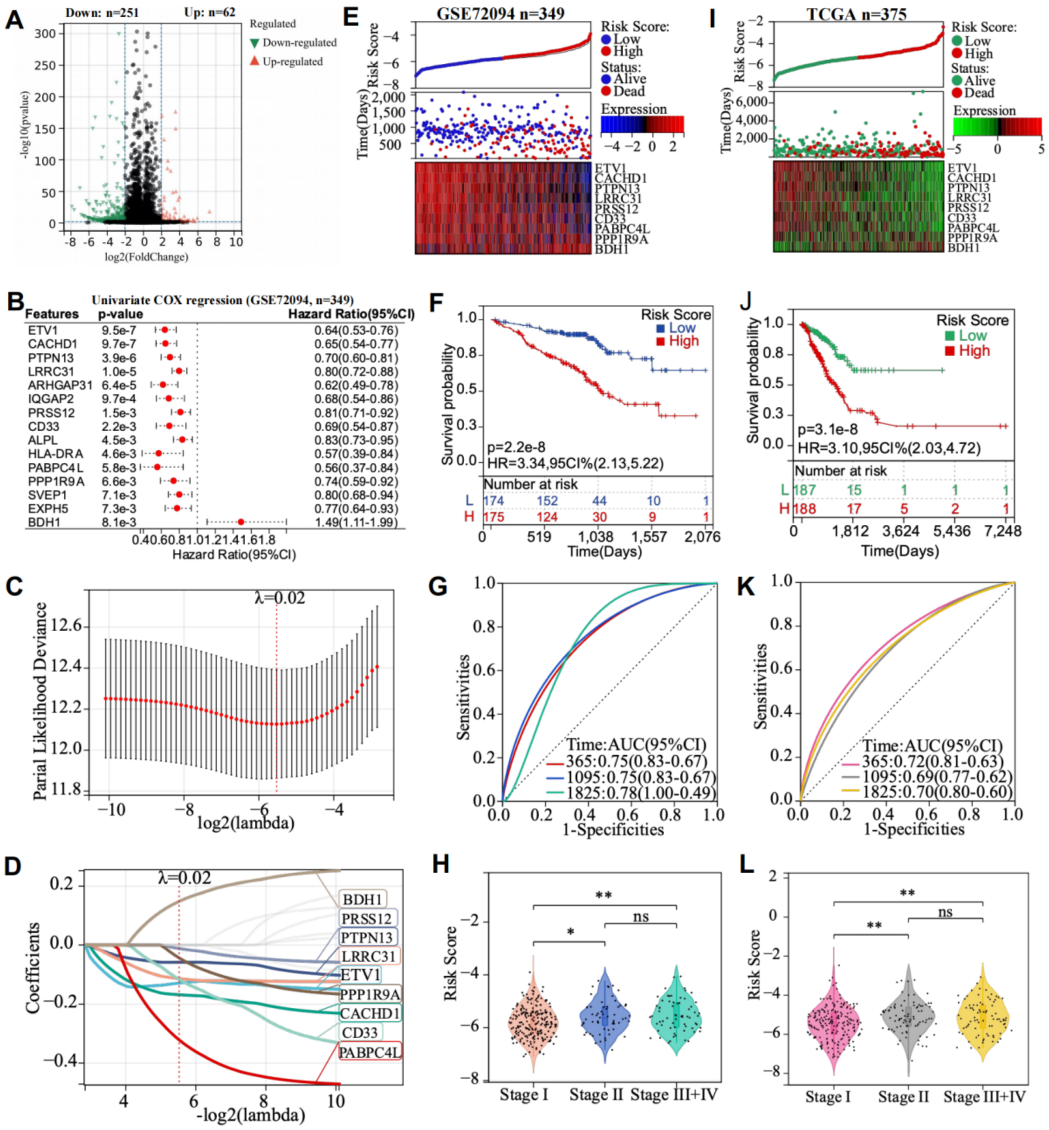

Figure S2 Establishing a prognostic model of BDH1 and its regulatory network. (A) The volcano plot showed differential genes between PC9 BDH1 KO#2 cells and control

cells. (B) The forest plot displayed a univariate Cox regression analysis of 15 prognosis-related genes. (C) Cross-validation showed that the optimal parameter in the LASSO model was 9. (D) Risk model was constructed using 9 prognostic genes screened by LASSO analysis. Analysis of prognostic models in GSE72095 (E-H) and TCGA-LUAD (I-L). (E, I) The prognostic heatmap presented the expression of 9 candidate genes in high- and low-risk groups. (F, J) Kaplan Meier curves showed the relationship between high- and low-risk LUAD patients and OS. (G, K) Time-dependent ROC curves based on risk scores at 1, 3, and 5 years were plotted. (H, L) Violin plots showed the distribution of risk scores of LUAD patients in different clinical stage groups (stage I, II, III+IV). (TCGA The Cancer Genome Atlas, DEGs Differentially expressed genes, AUC Area under the curve.)

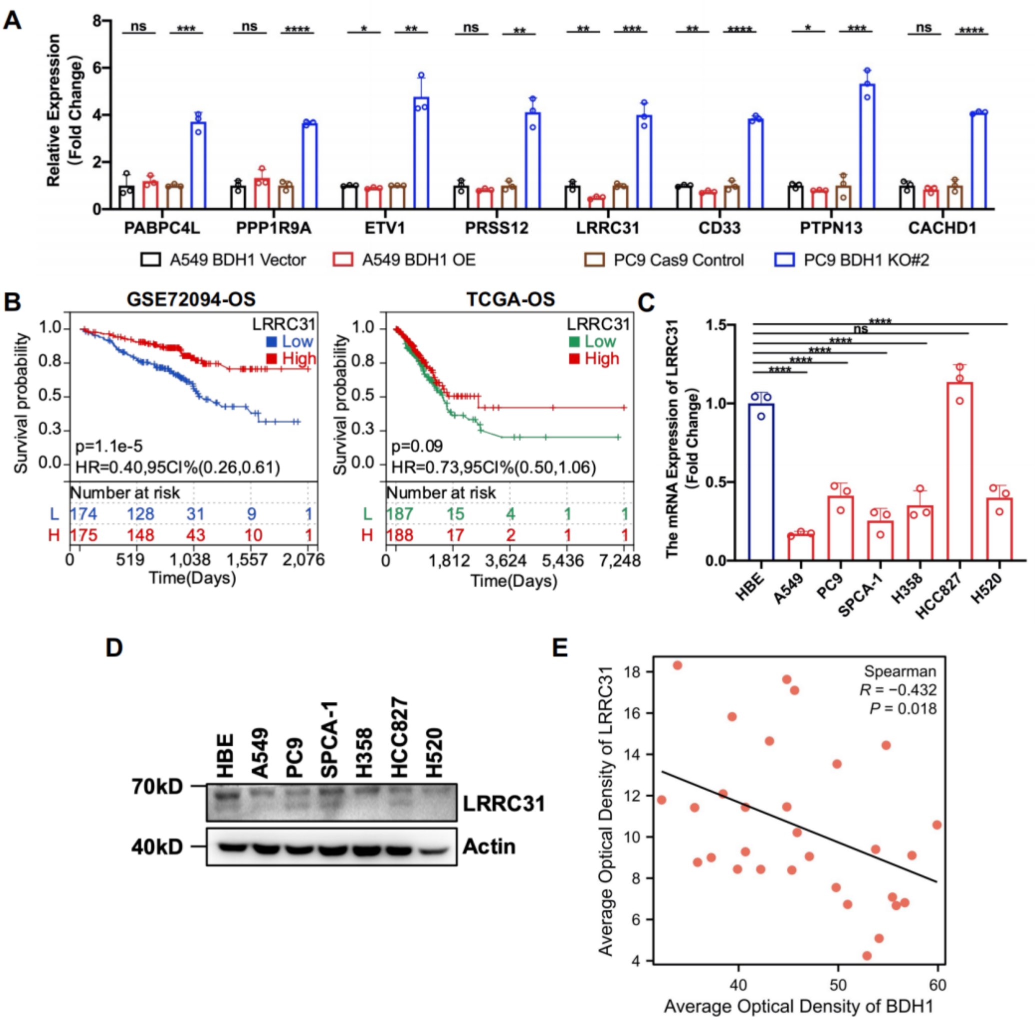

Figure S3 The expression of LRRC31 was negatively correlated with BDH1, and low expression of LRRC31 was significantly associated with poor prognosis in LUAD. (A) qRT-PCR detected the expression of eight candidate genes after overexpression of BDH1 and BDH1 knockout. (B) Survival curves showed the prognostic correlation of LRRC31 expression in GSE72094 and TCGA-LUAD with LUAD patients. (C, D) qRT-PCR and WB examined the expression level of LRRC31 in lung cancer cells and normal cells. (E) Correlation analysis detected the correlation between BDH1 and LRRC31 in LUAD tissues. (ns = no significant, * $P < 0.05$, ** $P < 0.01$, *** $P < 0.001$, **** $P < 0.0001$)

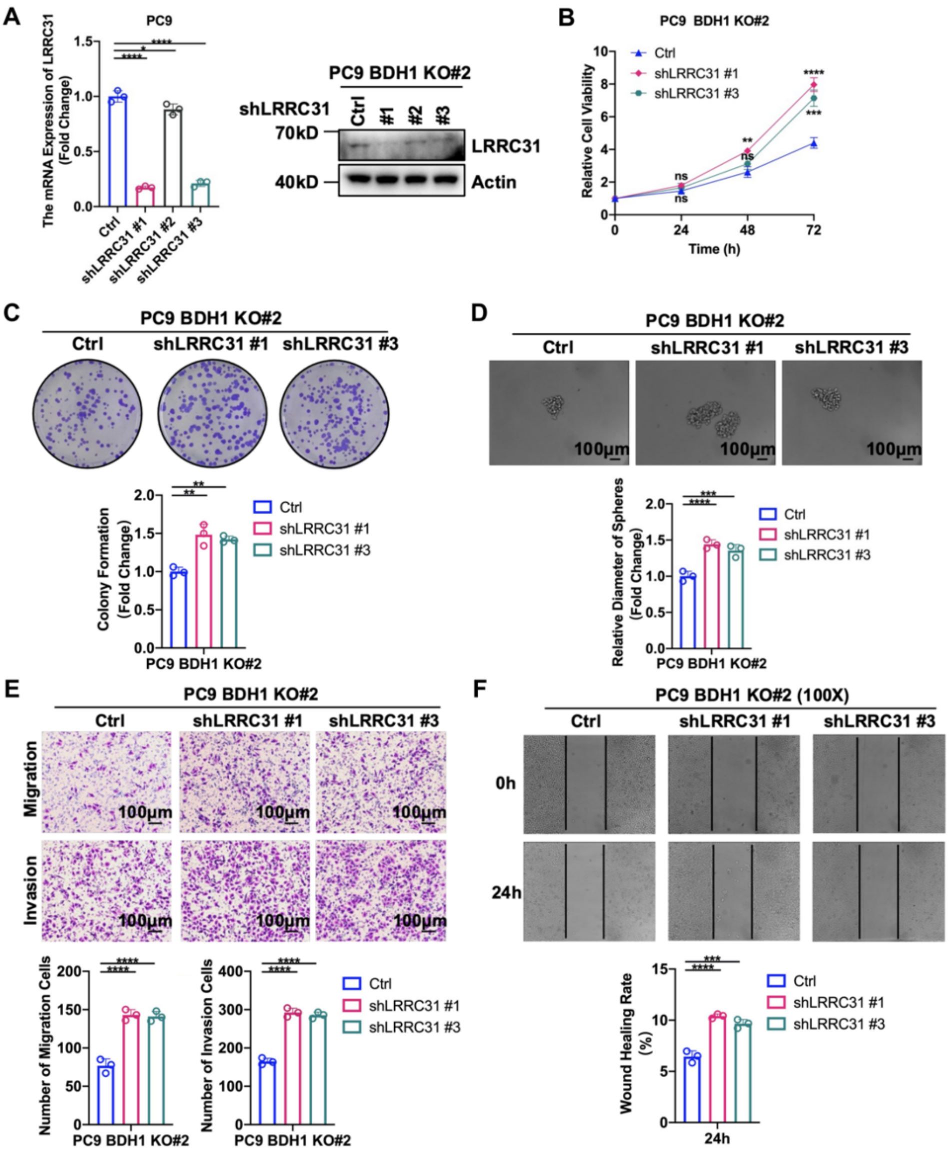

Figure S4 The significance of LRRC31 in LUAD regulation mediated by BDH1. (A) qRT-PCR and WB were employed to assess the knockdown efficiency of LRRC31 in PC9 BDH1 KO#2. (B) CCK-8 assay was used to demonstrate the effect of stable knockdown of LRRC31 on the proliferation of the PC9 BDH1 KO#2 cell line. (C) Clone formation assay was performed to detect the effect of stable knockdown of LRRC31 on the clonogenic ability of the PC9 BDH1 KO#2 cell line. (D) Tumor sphere formation assay detected the effect of stable knockdown of LRRC31 on the stem cell-like characteristics of the PC9 BDH1 KO#2 cell line. (E) Transwell migration and

invasion assay was used to demonstrate the effect of stable knockdown of LRRC31 on the migration and invasion of the PC9 BDH1 KO#2 cell line. (F) Wound healing assay was performed to test the effect of stable knockdown of LRRC31 on the wound healing ability of the PC9 BDH1 KO#2 cell line. (ns = no significant, * $P < 0.05$, ** $P < 0.01$, *** $P < 0.001$, **** $P < 0.0001$)

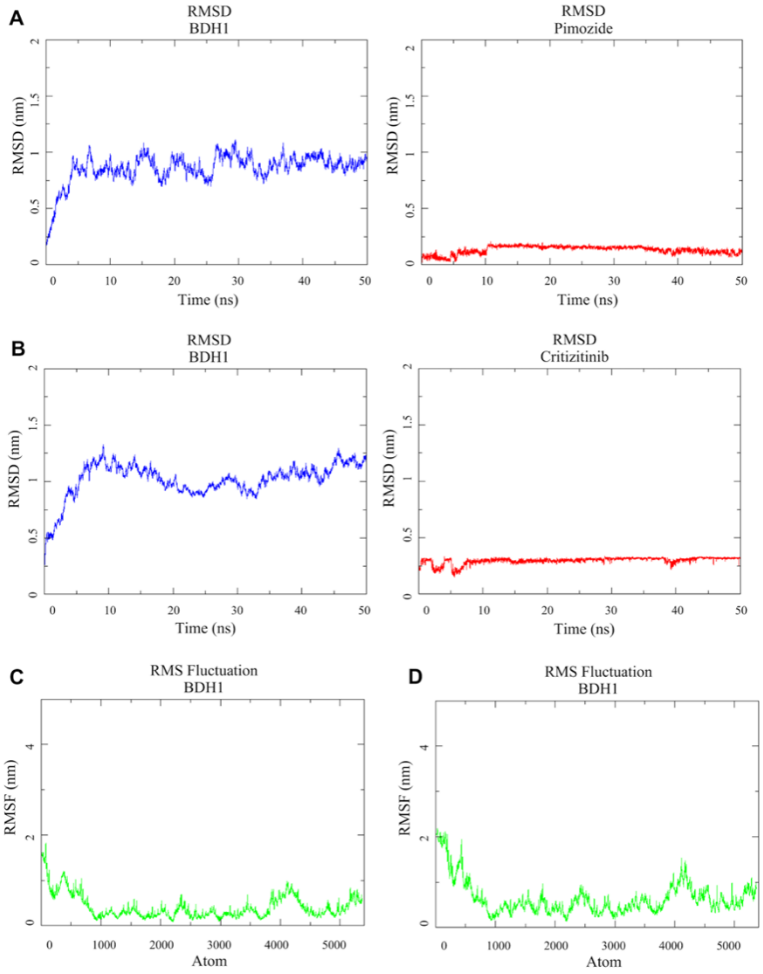

Figure S5 Molecular dynamics simulation of BDH1-pimozide and BDH1-crizotinib complex. (A, B) RMSD of BDH1-pimozide complexes and BDH1-crizotinib complexes in 50 ns MD simulations. (C, D) RMSF of BDH1-pimozide complexes and BDH1-crizotinib complexes in 50 ns MD simulations. (RMSD Root mean square deviation, RMSF Root mean square fluctuation)

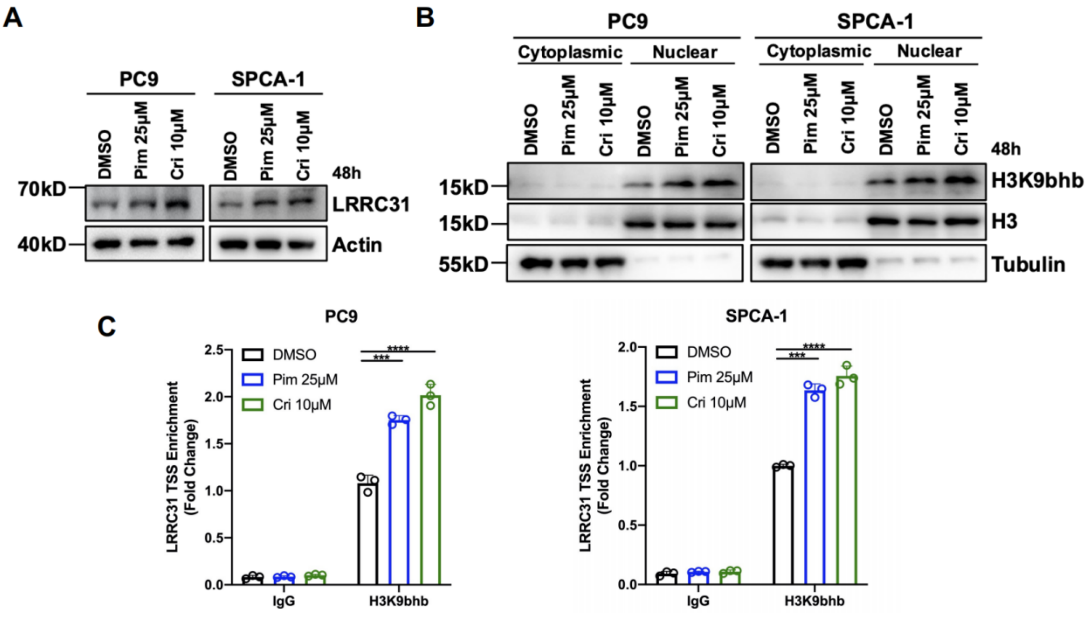

Figure S6 Pimozide and crizotinib targeting BDH1 inhibit BDH1/H3K9bhb/LRRC31 pathway in LUAD cells. (A) LRRC31 expression after pimozide or crizotinib concentration gradient and time gradient treatment of PC9 and SPCA-1. (B) The expression of H3K9bhb was assessed following treatment with a concentration gradient and time gradient of pimozide or crizotinib in PC9 and SPCA-1 cell lines. (C) ChIP assay was carried out to detect the enrichment of H3K9bhb in the TSS of LRRC31 in PC9 and SPCA-1 cells following exposure to pimozide or crizotinib. (*** $P < 0.001$, **** $P < 0.0001$)

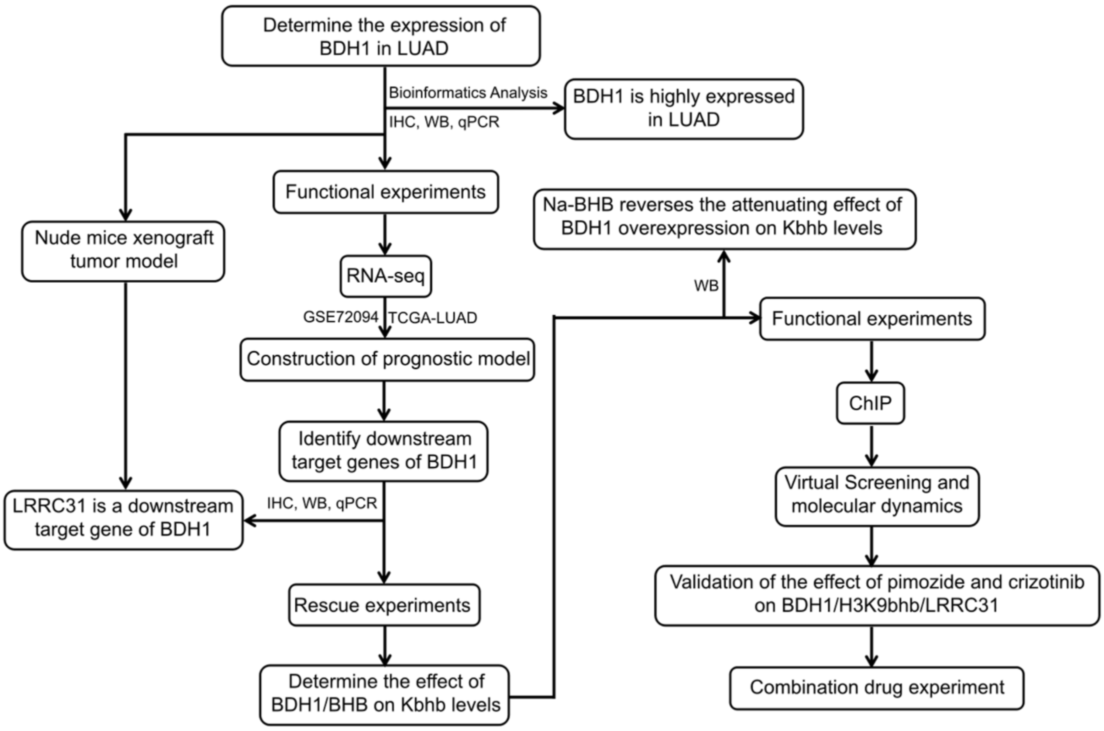

Figure S7 Workflow diagram.

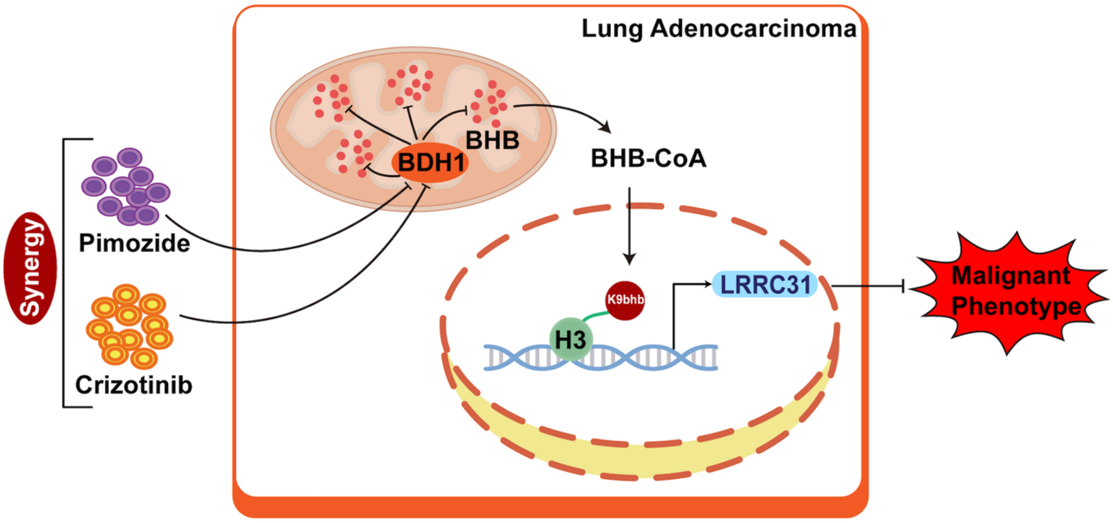

Figure S8 Illustration of BDH1 action in LUAD. BDH1 promotes LUAD development by regulating LRRC31 through H3K9bhb. Moreover, pimozide and crizotinib targeting BDH1 can synergistically inhibit the proliferation of LUAD.
